# NeuroRA: A Python Toolbox of Representational Analysis From Multi-Modal Neural Data

**DOI:** 10.3389/fninf.2020.563669

**Published:** 2020-12-23

**Authors:** Zitong Lu, Yixuan Ku

**Affiliations:** ^1^Guangdong Provincial Key Laboratory of Social Cognitive Neuroscience and Mental Health, Department of Psychology, Sun Yat-sen University, Guangzhou, China; ^2^Peng Cheng Laboratory, Shenzhen, China; ^3^Shanghai Key Laboratory of Brain Functional Genomics, Shanghai Changning-East China Normal University (ECNU) Mental Health Center, School of Psychology and Cognitive Science, East China Normal University, Shanghai, China

**Keywords:** representational similarity analysis (RSA), multivariate pattern analysis, multi-modal, Python, correlation analysis

## Abstract

In studies of cognitive neuroscience, multivariate pattern analysis (MVPA) is widely used as it offers richer information than traditional univariate analysis. Representational similarity analysis (RSA), as one method of MVPA, has become an effective decoding method based on neural data by calculating the similarity between different representations in the brain under different conditions. Moreover, RSA is suitable for researchers to compare data from different modalities and even bridge data from different species. However, previous toolboxes have been made to fit specific datasets. Here, we develop NeuroRA, a novel and easy-to-use toolbox for representational analysis. Our toolbox aims at conducting cross-modal data analysis from multi-modal neural data (e.g., EEG, MEG, fNIRS, fMRI, and other sources of neruroelectrophysiological data), behavioral data, and computer-simulated data. Compared with previous software packages, our toolbox is more comprehensive and powerful. Using NeuroRA, users can not only calculate the representational dissimilarity matrix (RDM), which reflects the representational similarity among different task conditions and conduct a representational analysis among different RDMs to achieve a cross-modal comparison. Besides, users can calculate neural pattern similarity (NPS), spatiotemporal pattern similarity (STPS), and inter-subject correlation (ISC) with this toolbox. NeuroRA also provides users with functions performing statistical analysis, storage, and visualization of results. We introduce the structure, modules, features, and algorithms of NeuroRA in this paper, as well as examples applying the toolbox in published datasets.

## Significance Statement

For the last two decades, neuroscience research envisions the prevalence of multivariate pattern analysis, in which representation similarity analysis (RSA) is one of the core methods. As representation bridges computation and implementation in David Marr's model, RSA bridges data from different modalities, including behavior, EEG, MEG, fMRI, et al. and even different species. Our toolbox NeuroRA is developed based on Python and can be applied for multi-modal neural data, as well as behavioral and simulated data. By calculating the representational dissimilarity matrix, neural pattern similarity, spatiotemporal pattern similarity, and inter-subject correlation with NeuroRA, we can assess representation similarities across datasets, subjects, space, and time. Statistical results can also be assessed by user-defined threshold and output to a data format that could be opened in other toolboxes.

## Introduction

In recent years, research on brain science based on neural data has shifted from univariate analysis toward multivariate pattern analysis (MVPA) (Norman et al., 2006). In contrast to the former, the latter accounts for the population coding for neurons. The decoding of neural activity can help scientists better understand the encoding process of neurons. As in David Marr's model, representation bridges the gap between a computation goal and implementation machinery (Marr, 1982). Representational similarity analysis (RSA) (Kriegeskorte et al., 2008) is an effective MVPA method that can successfully describe the relationship between representations of different data modalities, bridging gaps between humans, and animals. Therefore, RSA has been rapidly applied in investigating various cognitive functions, including perception (Evans and Davis, 2015; Henriksson et al., 2019), memory (Xue et al., 2010), language (Chen et al., 2016), and decision-making (Yan et al., 2016).

With technological development in brain science, various neural recording methods have emerged rapidly. Noninvasive methods that investigate brain activity such as electroencephalography (EEG), magnetoencephalography (MEG), functional magnetic resonance imaging (fMRI), and functional near-infrared spectroscopy (fNIRS) have been widely used for basic research. Meanwhile, invasive techniques such as electrocorticography (ECoG), stereo-electro-encephalography (sEEG), and some other neuroelectrophysiological methods have been applied to humans, non-human primates, and other animal species. The interpretation of results across different recording modalities becomes difficult. The RSA method, however, uses a representation dissimilarity matrix (RDM) to bridge data from different modalities. For example, studies have attempted to combine fMRI results with electrophysiological results (Kriegeskorte et al., 2008; Muukkonen et al., 2020), MEG results with electrophysiological results (Cichy et al., 2014), or behavioral results and fMRI results (Wang et al., 2018). Furthermore, with the rapid development of artificial intelligence (AI) (Jordan and Mitchell, 2015; Kriegeskorte and Golan, 2019), RSA can be used to compare representations in artificial neural networks (ANN) with brain activities (Khaligh-Razavi and Kriegeskorte, 2014; Yamins et al., 2014; Güçl and van Gerven, 2015; Eickenberg et al., 2017; Bonner and Epstein, 2018; Greene and Hansen, 2018; Kuzovkin et al., 2018; Urgen et al., 2019). In summary, RSA is a useful tool to combine the results of behavior and multi-modal neural data, leading to a better understanding of the brain. Even further, it can help us establish a clearer link between the brain and artificial intelligence.

Other existing tools for RSA include a module in PyMVPA (Hanke et al., 2009), a toolbox for RSA by Kriegeskorte (Nili et al., 2014), and an example in MNE-Python (Gramfort et al., 2013). However, they all have some shortcomings. MNE can only perform RSA for MEG and EEG data in one example. PyMVPA supports only basic functions, such as calculating the correlation coefficient and data conversion. Kriegeskorte's toolbox attached to their paper is designed mainly based on fMRI data, and users need to be proficient in MATLAB (Nili et al., 2014), which makes it difficult for users to apply to other datasets for EEG, MEG, or other types of data sources. We set to develop a comprehensive and universal toolbox for RSA, and Python was chosen as a suitable programming language. Python is a rapidly rising programming language having significant advantages for scientific computing (Sanner, 1999; Koepke, 2011). Because of its strong expansibility, it is more convenient to use Python for implementing a toolbox for representational analysis. NumPy (van der Walt et al., 2011), Scikit-learn (Pedregosa et al., 2011), and other extensions can execute and simplify various basic computing functions. Thus, some researchers select Python to develop toolkits in psychology and neuroscience, such as PsychoPy (Peirce, 2007) for designing psychological experiment programs, MNE-Python for EEG/MEG data analysis, and PyMVPA for utilizing MVPA in data from different modalities.

We have developed a novel and easy-to-use Python toolbox, NeuroRA (neural representational analysis), for comprehensive representation analysis. NeuroRA aims to use powerful computational resources with Python and conduct cross-modal data analyses for various types of neural data (e.g., EEG, MEG, fNIRS, fMRI, and other sources of neuroelectrophysiological data), as well as behavioral data and computer simulation data. In addition to the traditional functions of RSA, NeuroRA also includes specialized parts of representational analysis described in papers published on different research groups. These include neural pattern similarity (NPS) (Haxby, 2001; Cavanagh et al., 2018), spatiotemporal pattern similarity (STPS) (Xue et al., 2010; Lu et al., 2015), and inter-subject correlation (ISC) (Hasson et al., 2004). In the following sections, we detail the structure and function of NeuroRA and further apply it to several open datasets to guide users to use NeuroRA.

## Overview of Neurora

NeuroRA is an easy-to-use Python toolbox of representational analysis from multi-modal neural data. Users can apply NeuroRA to track the representation and compare representational similarity among different task conditions and modalities.

**Figure 1:**
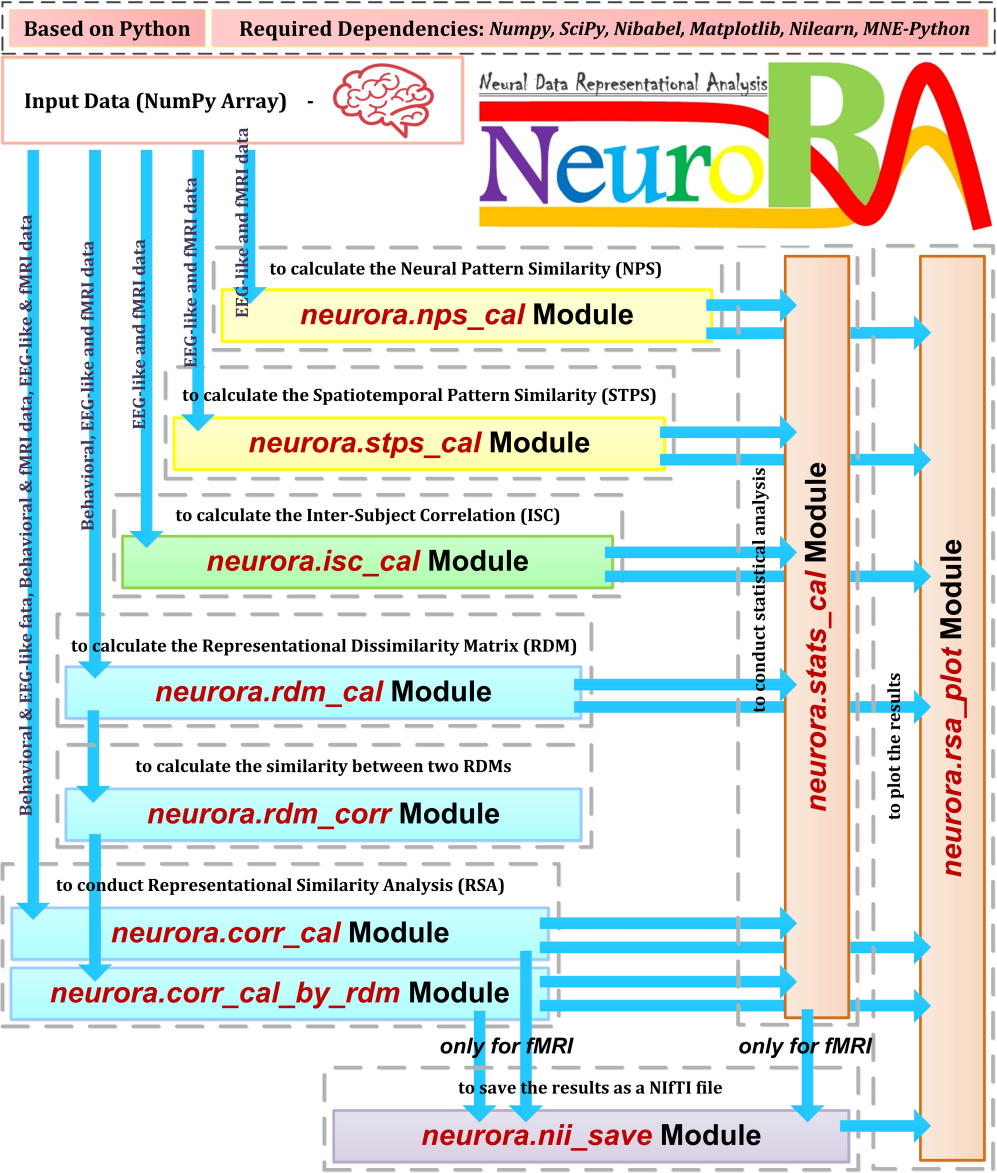
Overview of NeuroRA. NeuroRA is a Python-based toolbox and requires some extension packages, including NumPy, SciPy, Matplotlib, Nilearn, and MNE-Python. It contains several main parts: calculating neural pattern similarity (NPS), spatiotemporal pattern similarity (STPS), inter-subject correlation (ISC), and representation dissimilarity matrix (RDM), comparing representations among different modalities using RDMs, statistical analysis, saving results as a NIfTI file for fMRI data, and plotting the results. Each calculation part corresponds to one to two modules. The blue arrows indicate the feasible data flow.

The structure and features of NeuroRA are illustrated in Figure 1. It can analyze all types of neural (including EEG, MEG, fNIRS, fMRI, and other sources of neuroelectrophysiological data) and behavioral data. By utilizing the powerful computational toolbox in Python, NeuroRA gives users the ability to mine neural data thoroughly and efficiently.

NeuroRA provides abundant functions. First, NPS module reflects the correlation of brain activities induced under two different conditions. Second, STPS module reflects the representational similarity across different space and time points. Third, ISC module reflects the similarity of brain activities among multiple subjects under the same condition. Fourth, RDM module reflects the representation similarity between different conditions or stimuli with neural data from a given modality. Fifth, NeuroRA performs a correlation analysis between RDMs from different modalities to compare representations across modalities. This procedure can be applied according to different parameters; for example, the calculation can be applied for each subject, for each channel, for each time-point, or a combination of all of them.

In addition to calculating the above values, NeuroRA provides a statistical module to perform statistical analysis based on those values and a visualization module to plot the results, such as RDMs, representational similarities over time, and RSA-results for fMRI. Also, NeuroRA provides a unique approach to save the result of representational analysis back to the widely-used fMRI format NIfTI, generating a file obtained with user-defined output-threshold.

The required packages for NeuroRA include NumPy, SciPy (Virtanen et al., 2020), Matplotlib (Hunter, 2007), Nibabel (Brett et al., 2016), Nilearn, and MNE-Python, which are checked and automatically downloaded by installing NeuroRA. NumPy assists with matrix-based computation. SciPy helps with fundamental statistical analysis. Matplotlib and Nilearn are employed for the plotting functions. NiBabel is used to read and generate NIfTI files. MNE-Python is applied to load example MEG data in the demo. Users can download NeuroRA through only one line of command: *pip install neurora*. The website for our toolbox is https://neurora.github.io/NeuroRA/, and the website for online API documentation is https://neurora.github.io/documentation/. Additionally, GitHub URL for its source code is https://github.com/neurora/NeuroRA.

## Data Structures in Neurora

The calculations in NeuroRA are all based on multidimensional matrices, including deformation, transposition, decomposition, standardization, addition, and subtraction. The data type in NeuroRA is *ndarray*, an N-dimensional array class of NumPy. Therefore, users first convert their neural data into a matrix (*ndarray* type) as the input of NeuroRA, with information on the different dimensions of the matrix, such as the number of subjects, number of conditions, number of channels, and size of the image (see instructions in the software for details). Here, we give users some feasible methods for data conversion for different kinds of neural data in Supplemental Instructions for Data Conversion. The outputs of the functions in NeuroRA are square matrices with the same dimensions as the input matrix. The input and output data structures of key functions for calculation and statistical analysis in NeuroRA are shown in Supplementary Tables 1, 2.

## Neurora's Modules and Features

NeuroRA provides various functions to perform the representational analysis. Usually, data must be processed in multi-step ways, and this toolkit highly integrates these intermediate processes, making it easy to implement. In NeuroRA, only a simple function is required to complete the analysis. Users can obtain the required results after a necessary conversion of the data format.

Meanwhile, we attempt to add some adjustable parameters to meet the calculation requirements for different experiments and different modalities of data. Users can flexibly change the input parameters in the function to match their data format and computing goals.

NeuroRA includes the following core modules, and more modules could be added in the future or as requested.

*nps_cal*: A module to calculate the neural pattern similarity based on neural data.

*stps_cal*: A module to calculate the spatiotemporal pattern similarity based on neural data.

*isc_cal*: A module to calculate the inter-subject correlation based on neural data.

*rdm_cal*: A module to calculate RDMs based on multi-modal neural data.

*rdm_corr*: A module to calculate the correlation coefficient between two RDMs, based on different algorithms, including Pearson correlation, Spearman correlation, Kendall's tau correlation, cosine similarity, and Euclidean distance.

*corr_cal_by_rdm*: A module to calculate the representational similarities among the RDMs under different modes.

*corr_cal*: A module to conduct a one-step RSA between two different modes of data.

*nii_save*: A module to save the representational analysis results in a .nii file for fMRI.

*stats_cal*: A module to calculate the statistical results.

*rsa_plot*: A module to plot the results from the representational analysis. It contains the functions of plotting the RDM, plotting the graphs or hotmaps with results from the representational analysis by time sequence based on EEG or EEG-like (such as MEG) data, plotting the results of fMRI representational analysis (montage images and surface images).

## Representational Similarity Analysis Using Neurora

RSA has gradually become a popular method to explore information coding in the brain and computational models. Comparing whole dissimilarities among all task conditions in RDM, RSA becomes an effective approach to track the multidimensional representation among task conditions. On the one hand, researchers can construct hypothesis-based RDM for different conditions, then compare these theoretical models with RDMs from real neural activities to calculate how similar they are (Alfred et al., 2018; Feng et al., 2018; Hall-McMaster et al., 2019; Yokoi and Diedrichsen, 2019; Etzel et al., 2020). As a result, they can infer the information is coded in the brain. On the other hand, researchers can compare differences and similarities among multi-modal data by comparing the distance or correlation among RDMs computed using different data sources (Kriegeskorte et al., 2008; Cichy et al., 2014; Stolier and Freeman, 2016; Muukkonen et al., 2020). This cross-modal calculation has been increasingly used in comparing brain activities and deep neural networks during object processing (Khaligh-Razavi and Kriegeskorte, 2014; Yamins et al., 2014; Güçl and van Gerven, 2015; Eickenberg et al., 2017; Bonner and Epstein, 2018; Greene and Hansen, 2018; Kuzovkin et al., 2018; Urgen et al., 2019).

### Calculate One RDM or Multiple RDMs

**Figure 2:**
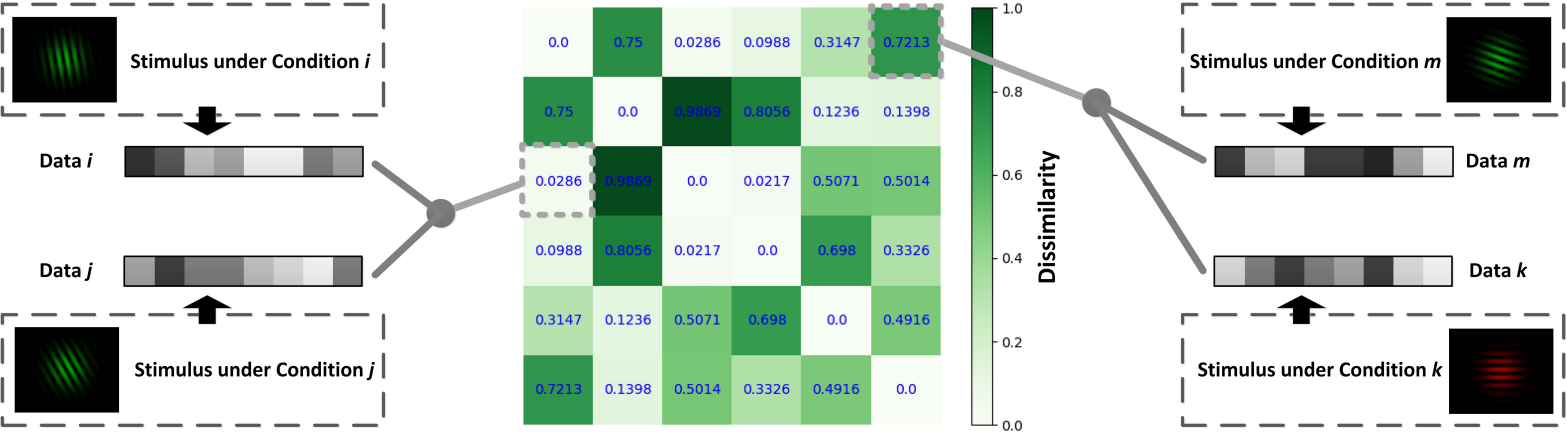
Schematic diagram for calculating the RDM. Different data can be obtained under different conditions. The value of the point [*i, j*] in RDM is obtained by calculating the dissimilarity (1-correlation coefficient *r*) between the data under condition *i* and that under condition *j*.

Constructing an RDM is a typical approach for comparing representations in neural data. By extracting data from two different conditions and calculating the correlations between them, we will obtain the similarity between the two representations under the two conditions. Subtract the obtained similarity index from 1 and get the values of the dissimilarity index in RDM (Figure 2). In Figure 2, different grating stimuli were observed to produce different neural responses, and the value in RDM presented the dissimilarity of neural activities between two different stimuli. As shown in the figure, the closer the two grating orientations were, the lower the dissimilarity. In a typical object recognition experiment, humans and monkeys were allowed to watch the same sets of visual stimuli (Kriegeskorte, 2008). Researchers calculated the humans' RDM based on fMRI data and the monkeys' RDM based on electrophysiological data. The results indicated that the neural patterns in RDMs were similar when humans and monkeys observed stimuli that belonged to the same category (animate or inanimate).

Assuming that the measured data from a certain condition under a total of *n* experimental conditions are denoted as *d*_1_, *d*_2_, …, *d*_*n*_, then the following RDM of *n*×*n* can be calculated by the corresponding function under the rdm_cal module from our toolkit:

$$\begin{array}{l} RDM=\left(\begin{array}{c} \begin{array}{c} \begin{array}{c} D_{11}\\ \vdots \\ D_{n1} \end{array}\;\begin{array}{c} D_{12}\\ \vdots \\ D_{n2} \end{array} \end{array}\;\begin{array}{c} \begin{array}{c} \cdots \\ \ddots \\ \cdots \end{array}\;\begin{array}{c} D_{1n}\\ \vdots \\ D_{nn} \end{array} \end{array} \end{array}\right) \end{array}$$

where *D*_*ij*_ denotes the dissimilarity between the data under condition *i* and that under condition *j*. The dissimilarity (*D*_*ij*_) is calculated as follows:

$$\begin{array}{l} D_{ij}=1-similarity\left(d_{i},d_{j}\right) \end{array}$$

**Figure 3:**
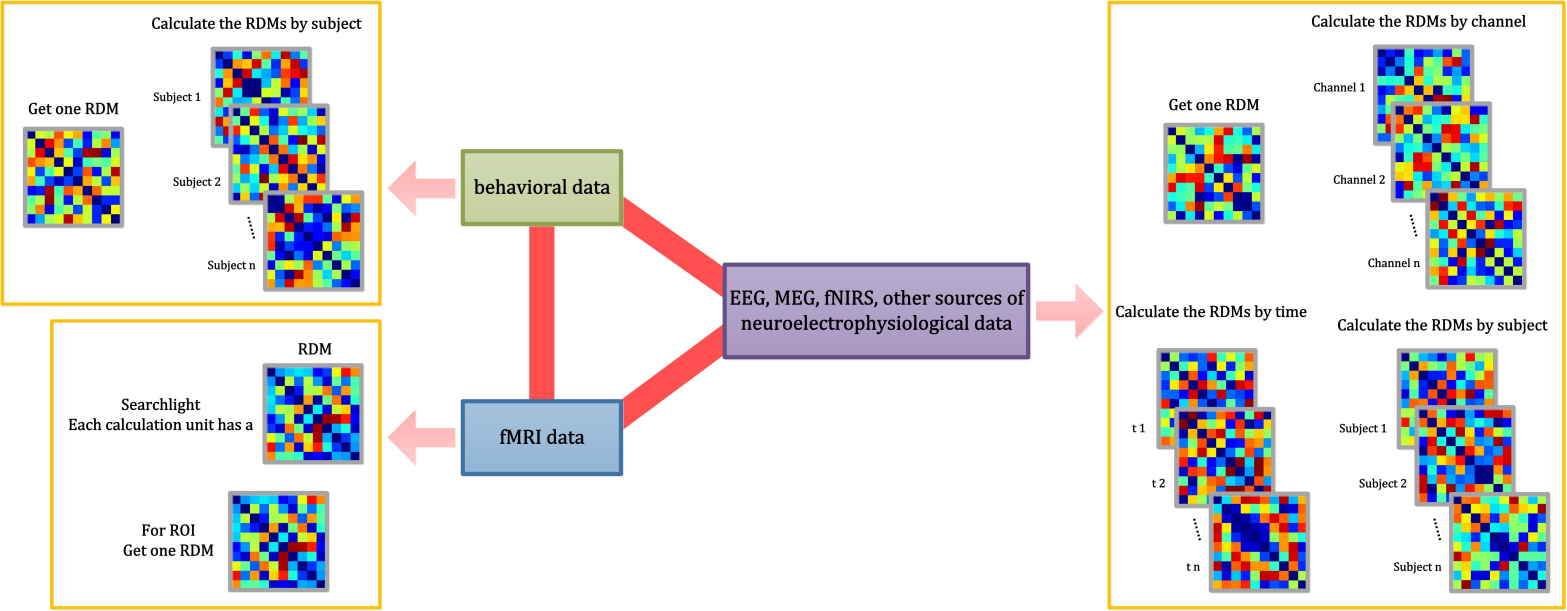
RDM calculations implemented in NeuroRA. NeuroRA is capable of calculating an RDM using data from different modes, including behavior, fMRI, EEG, MEG, fNIRS, and other sources of neuroelectrophysiological data. The red bold lines denote the ability to perform calculations between two modes. The pink arrow denotes the alternative calculation methods of the corresponding mode.

When computing the RDM, multiple measures are provided in NeuroRA, including correlation distance (based on Pearson correlation), Euclidean distance, and Mahalanobis distance. All functions in *neurora.rdm_cal* module has a parameter named *method*, which can be set to change the measure you want (default is for computing based on correlation distance). The application of calculating RDMs is not restricted. NeuroRA can perform computations based on multiple modal neural data from behavioral data to brain imaging data (Figure 3).

In certain cases, researchers must calculate RDM separately for each participant, or they may calculate RDM independently for each channel or each time point (Hall-McMaster et al., 2019; Henriksson et al., 2019). We resolve these issues in NeuroRA by providing several input parameters in the functions that allow users to make the corresponding changes to get one RDM or multiple RDMs by different subjects or channels or time-windows or searchlight (for fMRI) or specific ROI (for fMRI) (Figure 3). Users can change the calculation parameters according to their requirements, and consequently, the corresponding output formats change. Detailed instruction of the shape of the input, the parameter settings with calculation implementation, the corresponding shape of the output, and recommended next steps can be seen in Supplementary Table 1.

### Representational Analysis Among Different RDMs

#### Analysis Between Two RDMs

NeuroRA provides a convenient way to calculate cross-modal similarity by computing the similarities between two RDMs from different modalities. We offer several solutions to calculate the similarity (or correlation coefficient), including Pearson correlation, Spearman correlation, Kendall's tau correlation, cosine similarity, and Euclidean distance. Users can freely change parameters to select different computing methods.

**Figure 4:**
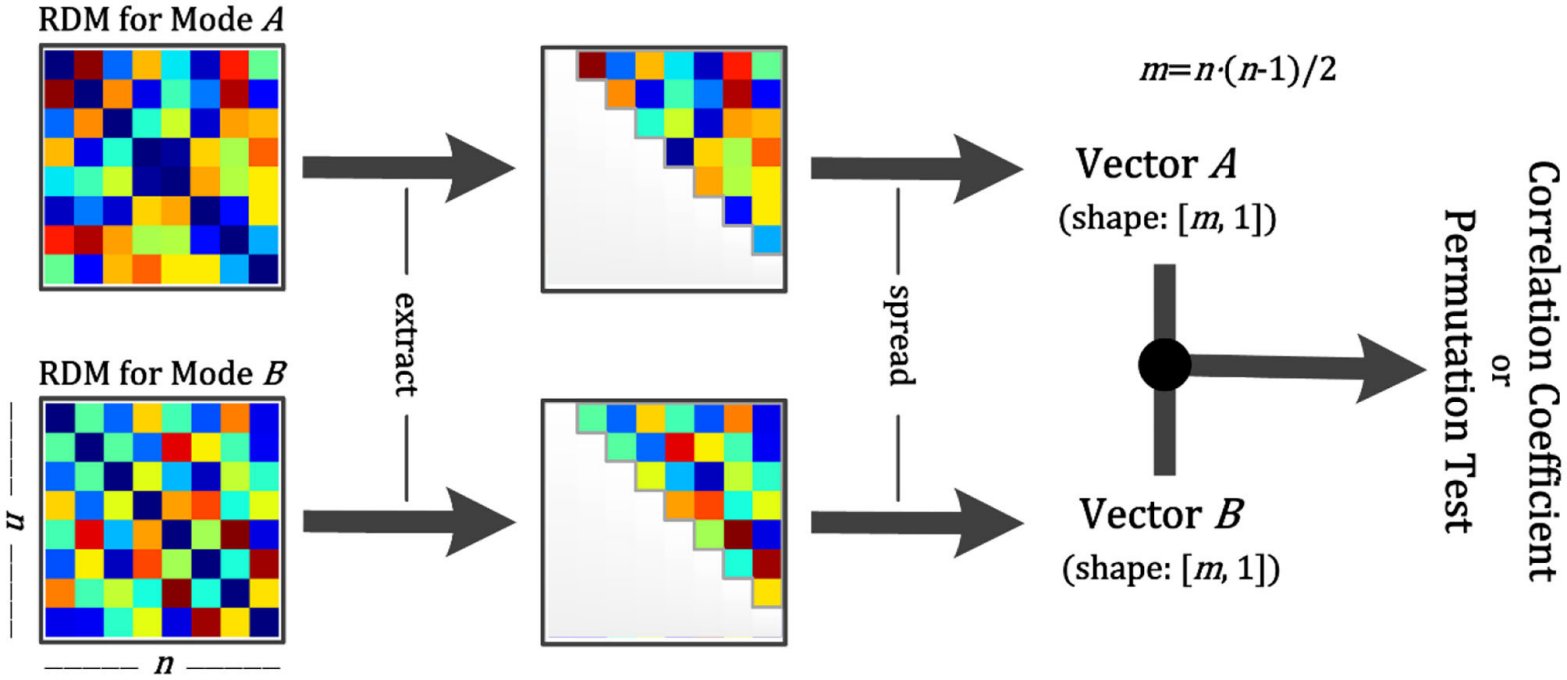
Schematic diagram for calculation between two RDMs. Step 1: Obtain two RDMs from different modal data. Step 2: Extract the points of the upper diagonal (within the gray line). Step 3: Spread them as vectors. Step 4: Calculate the correlation coefficient or conduct a permutation test between two vectors. The former returns the correlation coefficient and the *p*-value, and the latter returns only the *p*-value.

For the calculations, we first reshape the square matrices into vectors and then calculate similarities (Figure 4). Previous studies calculated the correlation coefficient between two RDMs using the diagonal values, making the result deceptively high (Ritchie et al., 2017). We avoid this by removing the diagonal values and include only half of the matrix to reduce the duplication, as the upper and lower halves of the RDM are identical (Figure 4).

Furthermore, NeuroRA provides a permutation test to determine whether the two RDMs are related. The permutation test is based on the random shuffling of data and is suitable for data with a small sample size (Efron and Tibshirani, 1994). We first shuffle the values in the two RDMs and re-calculate the similarity matrix between the two RDMs. By repeating this procedure 5000 times (the number of iterations here can be defined by users), we get the final *p*-values from this permutation distribution.

#### Analysis Among Multiple RDMs

**Figure 5:**
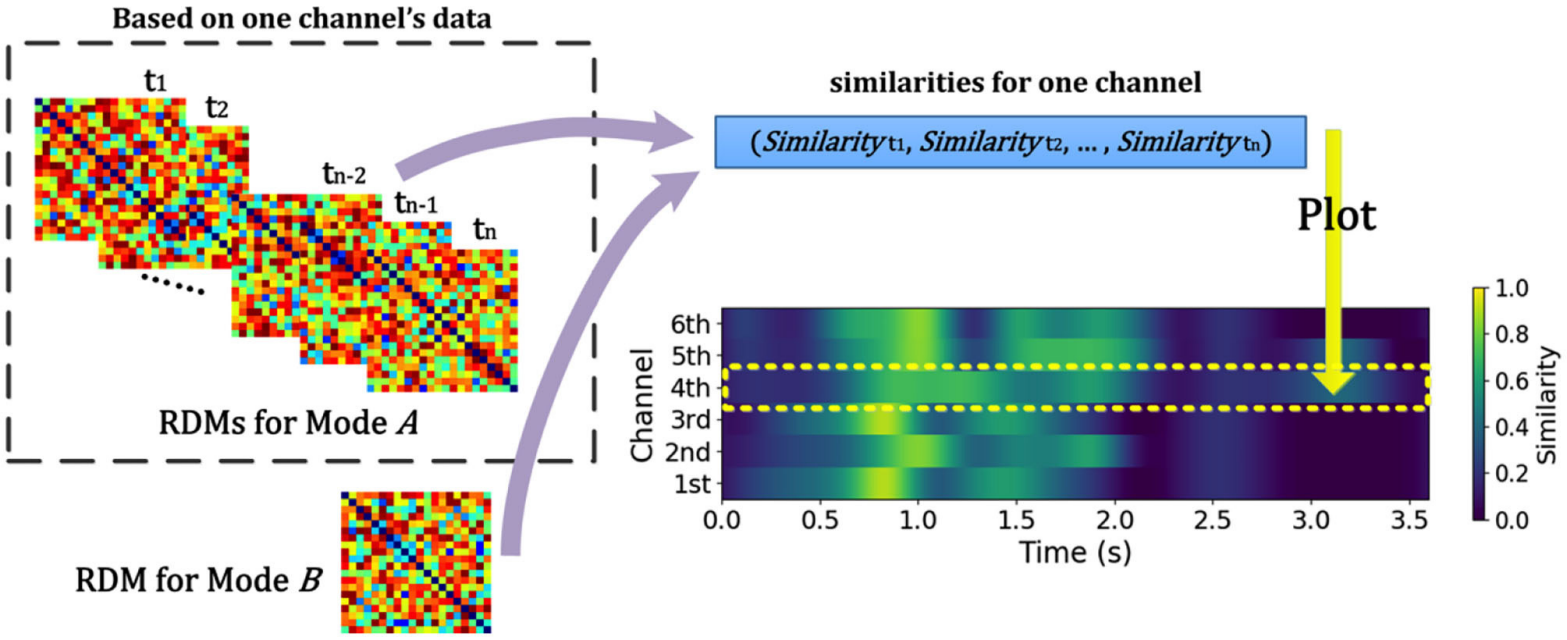
Schematic diagram for calculating similarities between RDM from different modes across time and channel for EEG and EEG-like (such as MEG or sEEG) data. NeuroRA calculates the similarities between RDMs for mode A (EEG and EEG-like data) and one RDM for mode B (such as behavior). Such calculation can be performed across each time-window and each channel. Each value in time-channel result-image (bottom right) corresponds to a similarity index (for example, the Pearson correlation) between RDMs from two Modes.

NeuroRA can also perform calculations based on multiple RDMs, rather than only two RDMs corresponding to two modalities. Consequently, we can expand it to conditions with multiple RDMs from different modalities. For instance, when you obtain a behavioral RDM from behavioral data and wish to compare it with other modal data, a problem may arise as more than one RDM can be obtained based on other modal data, such as EEG or fMRI. Our toolbox provides “searchlight” computation to perform correlation analysis between RDM from one mode (behavior, or any of neural data) and RDM from other modes (each brain region, time interval, or others) one by one (Figure 3). For example, calculations based on EEG data can obtain one RDM per channel or time interval or both (Figure 5). Table 1 is a script example for using NeuroRA to calculate the similarities between behavioral RDM and EEG RDMs per channel by time sequence. Another simple example is when users want to see which brain regions are highly correlated with the behavioral performance or a specific coding model, they can get one behavioral or model RDM based on behavioral response time or accuracy, and they may also get many fMRI RDMs from different regions. Users can put these two kinds of RDMs (behavioral or model RDM and fMRI RDMs) into our function, and they will get results showing the regions that are highly correlated with behavioral or model patterns based on thresholds of significance (*p*-value) or correlation values set by users (**Table 3**, more details on fMRI calculation are described in the next section). These convenient functions of ergodic computation cover the vast majority of cross-modal research needs.

**Table 1:** Scripts of representational analysis between behavioral data and EEG data for each channel in NeuroRA.

Scheme 1	1	from neurora.rdm_cal import bhvRDM, eegRDM
	2	from neurora.corr_cal_by_rdm import rdms_corr
	3	
	4	# calculate the behavioral RDM for each subject
	5	# the shape of bhv_data should be [n_conditions, n_subjects, n_trials]
	6	# the shape of bhv_rdms will be [n_subjects, n_conditions, n_conditions]
	7	bhv_rdms = bhvRDM(bhv_data, sub_opt=1)
	8	
	9	# calculate the eeg RDMs for each channel & each subject
	10	# the shape of eeg_data should be [n_conditions, n_subjects, n_trials, n_channels, n_times]
	11	# the shape of eeg_rdms will be [n_subjects, n_channels, n_conditions, n_conditions]
	12	eeg_rdms = eegRDM(eeg_data, sub_opt=1, chl_opt=1)
	13	
	14	# initialize the correlation coefficients
	15	corrs = np.zeros([n_subjects, n_channels, 2], dtype=np.float)
	16	
	17	# calculate the correlation coefficients between behavioral RDM and eeg RDMs
	18	# the shape of corrs is [n_subjects, n_channels, 2], 2 represents a *r*-value & a *p*-value
	19	for sub in range(n_subjects):
	20	corrs[sub] = rdm_corr(bhv_rdms[sub], eeg_rdms[sub])
Scheme 2	21	from neurora.corr_cal import bhvANDeeg_corr
	22	
	23	# calculate the correlation coefficients between behavioral RDM and eeg RDMs
	24	corrs = bhvANDeeg_corr(bhv_data, eeg_data, sub_opt=1, chl_opt=1)

*Users can input data from different modes and obtain the correlation between the results of the two modes. If users want to conduct the calculation for each time-windows, they can set the parameters: time_opt, time_win & time_step in function eegRDM and bhvANDeeg_corr*.

To simplify users' experience, our toolbox offers a one-step option between different modes (Table 1 Scheme 2 is a one-step example for calculating a similarity index between behavior and EEG). Users can input data from two modalities, and the toolbox will directly return the final results of representation analysis. It is very convenient and efficient when users do not need to obtain the RDMs in the intermediate stages. Thus, users can use two modules, *corr_cal* and *corr_cal_by_rdm*, to calculate the representational similarity between two different modalities. The former module provides the calculation based on data from two different modalities. The later module provides the calculation based on RDMs after previous computing from two modalities' data. In both modules for calculating cross-modal similarity, users can set different parameters to meet the requirements under different conditions (calculate for each channel, etc.). More detailed instructions of the shape of the input, the parameter settings with calculation implementation, the corresponding shape of the output, and recommended next steps about these modules are shown in Supplementary Table 1.

### Representational Analysis for fMRI

**Figure 6:**
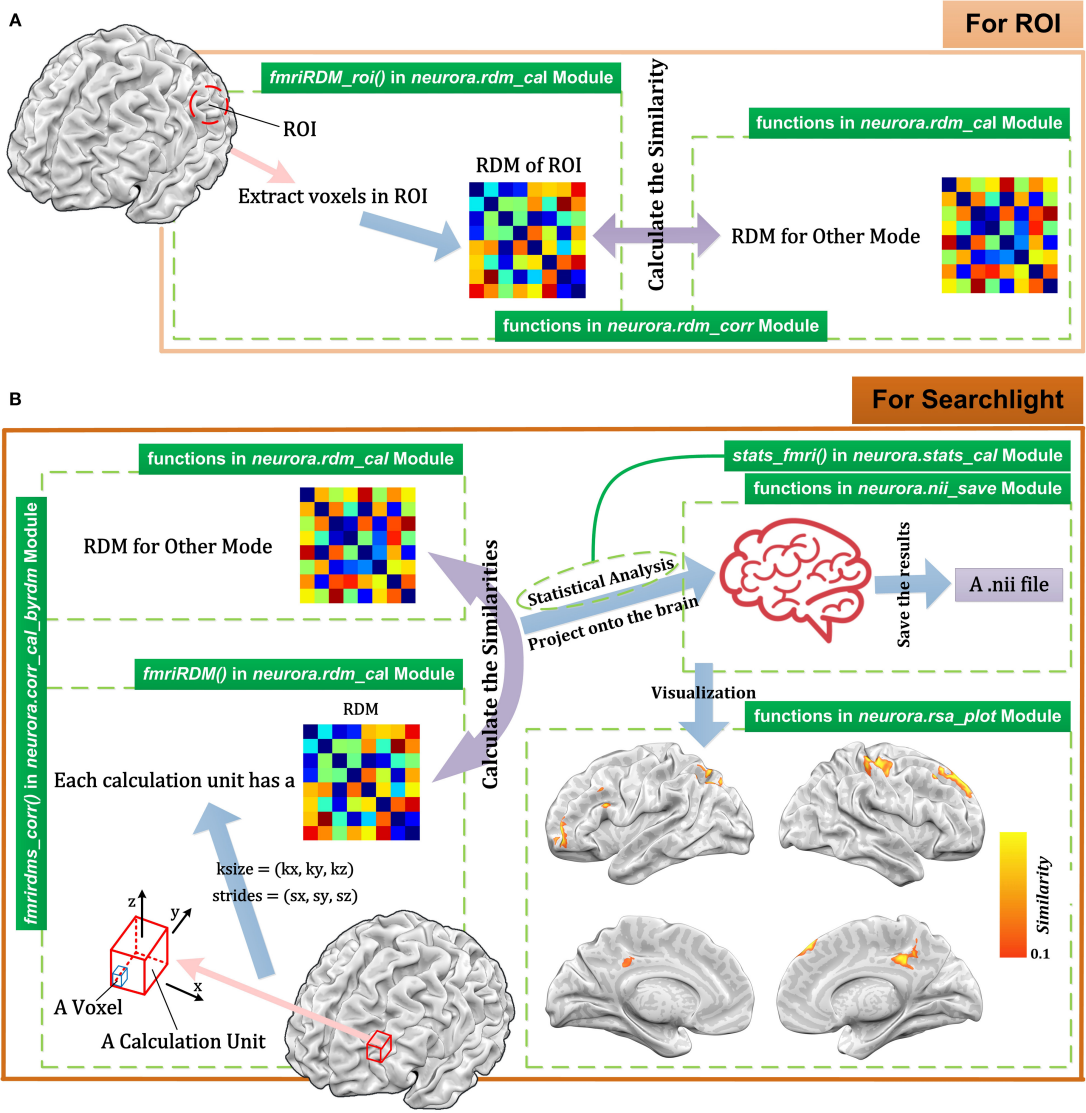
Schematic diagram for representational analysis for fMRI data using NeuroRA. **(A)** The calculating process for ROI-based analysis. For each ROI, users can calculate the RDM based on the voxels in ROI and get the similarity between ROI RDM and the RDM for other modes. **(B)** The calculating process for searchlight-based analysis. For each searchlight step, users define the size and strides of the calculation unit. After computations between the RDMs within the searchlight blob for fMRI and the RDM for other modes (e.g., behavioral data, computer-simulated data), a NIfTI file can be obtained. At the bottom right is a demo of the resulting NIfTI file drawn with NeuroELF (http://neuroelf.net), and color-coded regions indicate the strength of representation similarity between two modes. The green text on the green indicates which function to use for the corresponding step.

fMRI is a largely used method in cognitive neuroscience. In the RSA of fMRI data (Johnson et al., 2005; Poldrack, 2012; Rosen and Savoy, 2012; Lawrence et al., 2019), researchers typically wish to calculate RDMs for different brain regions. In NeuroRA, users can conduct representational analysis using ROIs or searchlight across the whole brain (Figure 6).

#### ROI-Based Computation

For ROI-based computation, users are required to input both fMRI data and a 3-D mask matrix whose size should be consistent with the size of the fMRI image corresponding to fMRI data. The valid voxels which belong to ROI are extracted, and different activities under different conditions of these voxels are spread out as vectors. Then the ROI-based RDM can be calculated by computing the dissimilarities among these vectors. Finally, we can calculate the similarity between this ROI-based RDM and the RDM for another modality. Steps for ROI-based computation with corresponding functions in NeuroRA are shown in Figure 6A.

#### Searchlight-Based Computation

Searchlight related functions in NeuroRA provide rich parameters for user customization. For each searchlight step, users can customize the size of the basic calculation unit [*k*_*x*_, *k*_*y*_, *k*_*z*_]. Each *k* indicates the number of voxels along the corresponding axis. The strides between different calculation unit must be decided as [*s*_*x*_, *s*_*y*_, *s*_*z*_]. The strides refer to how far the calculation unit is moved before another computation is made. Each *s* indicates how many voxels exist between two adjacent calculation units along the corresponding axis. For the fMRI data of size [*X, Y, Z*], the kernel size is usually set to be more than one voxel so that each voxel can exist in multiple kernels (calculation units). Therefore, *N* computations are required here:

$$\begin{array}{l} N=(\left\lfloor \frac{\left(X-k_{x}\right)}{s_{x}}\right\rfloor +1)\times (\left\lfloor \frac{\left(Y-k_{y}\right)}{s_{y}}\right\rfloor +1)\\ \;\times (\left\lfloor \frac{\left(Z-k_{z}\right)}{s_{z}}\right\rfloor +1) \end{array}$$

This implies that *N* RDMs must be calculated, which are each related to the corresponding calculation unit. After obtaining searchlight RDMs, users can calculate the similarities between fMRI and other modes. In NeuroRA, the final correlation coefficient of one voxel is the mean value of the correlation coefficients calculated by all kernels that contain this voxel.

Figure 6B shows the steps for searchlight-based computation with corresponding functions in NeuroRA. Table 2 is a script demo to understand how to conduct a searchlight-based analysis for fMRI data. We could first calculate the fMRI RDMs within each searchlight blob and then obtain similarities between fMRI RDMs and a behavioral RDM or a coding model RDM, which is constructed based on the hypothesis all over the whole brain. In a hypothesis-based RDM, values corresponding to the same condition have the highest similarity, and values corresponding to different conditions have a low similarity.

**Table 2:** Script of searchlight representational analysis between fMRI data and a coding model in NeuroRA.

1	from neurora.rdm_cal import fmriRDM
2	from neurora.corr_cal_by_rdm import fmrirdms_corr
3	import numpy as np
4	
5	# calculate the searchlight fMRI RDMs for each subject
6	# the shape of fmri_data should be [n_conditions, n_subjects, nx, ny, nz]
7	# nx, ny, nz represent the size of fMRI-img
8	# here, the size of calculation unit is [3, 3, 3] and the strides for calculating is [1, 1, 1]
9	# the shape of fmri_rdms will be [n_subjects, n_x, n_y, n_z]
10	# n_x, n_y, n_z represent the number of calculation units for searchlight along the x, y, z axis.
11	fmri_rdms = fmriRDM(fmri_data, ksize=[3, 3, 3], strides=[1, 1, 1], sub_opt=1)
12	
13	# initialize the correlation coefficients
14	corrs = np.zeros([n_subjects, n_x, n_y, n_z, 2], dtype=np.float)
15	
16	# calculate the correlation coefficients between searchlight fMRI RDMs and a model RDM
17	# the shape of model_rdm should be [n_conditions, n_conditions]
18	# the shape of corrs will be [n_subjetcs, n_x, n_y, n_z, 2], 2 represents a *r*-value & a *p*-value
19	for sub in range(n_subjects):
20	corrs[sub] = fmrirdms_corr(model_rdm, fmri_rdms[sub])

*The calculation parameters of fMRI data are ksize=[3, 3, 3] and strides=[1, 1, 1]. Users can just input different data and obtain the correlation results between two modes*.

#### Save Results as a NIfTI File

NeuroRA provides two functions in *nii_save* module, *corr_save_nii*() and *stats_save_nii*(), to save the similarity result or the statistical result as a NIfTI file with thresholding parameters as well. These two functions are used similarly. The former function is used for saving the results of *r*-values after calculating the similarities between fMRI mode and another mode. The latter function is used for saving the results of *t*-values after statistical analysis. Table 3 is a script to help users understand how to use *corr_save_nii*() to save the similarity results as a NIfTI file. Users can set certain thresholds for *p*-values, *r*-values (only in *corr_save_nii*() function) or *t*-values (only in *stats_save_nii*() function). Also, users can select Family-Wise-Error (FWE) or False-Discovery-Rate (FDR) correction methods to control for multiple comparisons across the whole brain. Furthermore, users can choose whether to smooth the results, whether to plot automatically, etc. For example, if the threshold for *p*-value is set as 0.05, the final NIfTI file returned will be filtered with *p* < 0.05, and all voxels with *p*>=0.05 will be set as 0.

**Table 3:** Script of saving the calculation results as a NIfTI file for fMRI data.

1	from neurora.nii_save import corr_save_nii
2	
3	# corrs represents the similarities (correlation coefficients) between fMRI and other mode
4	# the shape of corrs should be [n_x, n_y, n_z, 2]
5	# filename represents the filename of the result.nii file
6	# affine represents the information of the fMRI-image array data in a reference space
7	# here, the size of fMRI-image is [60, 60, 60], the size of calculation unit is [3, 3, 3] and the
8	# strides for calculating is [1, 1, 1]
9	filename = “demo_result.nii”
10	corr_save_nii(corrs, filename, affine, size=[60, 60, 60], size=[60, 60, 60], ksize=[3, 3, 3],
11	strides=[1, 1, 1], p=0.05, correct_method='FDR')
12	
13	# The output is an [60, 60, 60] NumPy-array
14	# And a.nii file named 'demo-results.nii' has been generated

*Users can get the correlation results based on the script in Table 2. The NIfTI file can be obtained by entering some necessary parameters*.

### Other Representational Analysis

In addition to RSA, users can conduct the analysis of NPS, STPS, and ISC with NeuroRA. Detailed implementation of these analysis methods can be seen in Supplementary Information for the Implementation of Analysis Methods. Our toolkits have separate modules to conduct these calculations (Table 4). Just like RSA from multiple modalities, the calculations for these other representational analysis methods support EEG-like data as well as fMRI data. Users can calculate the results for each channel or region, each time-window from a time series, each ROI or searchlight blobs (for fMRI) as they wish by selecting different functions and setting specific parameters. These calculations are used in a similar way to calculate RDM or RSA, as described in the above sections. In detail, Supplementary Table 1 shows the shape of the input, the parameter settings with calculation implementation, the corresponding shape of the output, and recommended next steps in the analysis. Additionally, users can use *help*() (a built-in function in Python) to see and understand the detailed description of the purpose of the specific function or module.

**Table 4:** Modules and functions for NPS, STPS, ISC in NeuroRA.

**Analysis Method**	**Module for Computing**	**Functions for Statistical Analysis**
NPS	*neurora.nps_cal* module	For EEG-like: *neurora.stats_cal.stats*() For fMRI: *neurora.stats_cal.stats_fmri*()
STPS	*neurora.stps_cal* module	For EEG-like: *neurora.stats_cal.stats_stps*() For fMRI: *neurora.stats_cal.stats_stpsfmri*()
ISC	*neurora.isc_cal* module	For EEG-like: *bneurora.stats_cal.stats*() For fMRI like: *neurora.stats_iscfmri*()

### Statistical Analysis

NeuroRA provides functions for statistical analysis based on the representational analysis results. The inputs are the similarity maps for each subject, which can be obtained by functions in calculation modules (*corr_cal, corr_cal_by_rdm, nps_cal, stps_cal*, and *isc_cal* modules), and the output will be the statistical results (a *t*-value & *p*-value map) (Table 5). The output from the functions of calculation modules always includes an *r*-value map and a *p*-value map. Although only the *r*-value map is used for subject-level statistical analysis, users can directly input the output of functions in calculation modules as the input of functions in *stats_cal* module for convenience.

**Table 5:** Example of statistical analysis for channel-time based EEG RSA calculation and searchlight fMRI RSA calculation.

**Type of Calculation**	**Example Script**
*channel-time based EEG-like calculation*	from neurora.corr_cal import bhvANDeeg_corr from neurora.stats_cal import stats # calculate the correlation coefficients between behavioral data and EEG data corrs=bhvANDeeg_corr(bhv_data, eeg_data, sub_opt=1, chl_opt=1, time_opt=1) # the shape of corrs should be [n_subs, n_chls, n_ts, 2] stats(corrs, permutation=True, iter=1000) # The output is an [n_chls, n_ts, 2] NumPy-array # 2 represents a *t*-value and a *p*-value
*searchlight fMRI calculation*	from neurora.corr_cal import bhvANDfmri_corr from neurora.stats_cal import stats_fmri # calculate the correlation coefficients between behavioral data and fMRI data corrs=bhvANDfmri_corr(bhv_data, eeg_data, sub_opt=1, chl_opt=1, time_opt=1) # the shape of corrs should be [n_subs, n_x, n_y, n_z, 2] stats_fmri(corrs, permutation=True, iter=10000) # The output is an [n_x, n_y, n_z, 2] NumPy-array

In this part, the correlation coefficients calculated by calculation modules are tested against zero for significance. Besides, we add a permutation test to all processes of statistical analysis. This means the statistical significance could be assessed through a permutation test by randomly shuffling the data and calculated the results for many iterations (for example, 5000) to draw a distribution. Real data exceeding 95% of the distribution are regarded as significant. Table 5 is a script to show how to use *stats_cal* module to conduct statistical analysis for RSA results from different modes. Users can independently choose to use the permutation test or not and change the iteration number by set parameters in related functions.

### Visualization of Results

**Figure 7:**
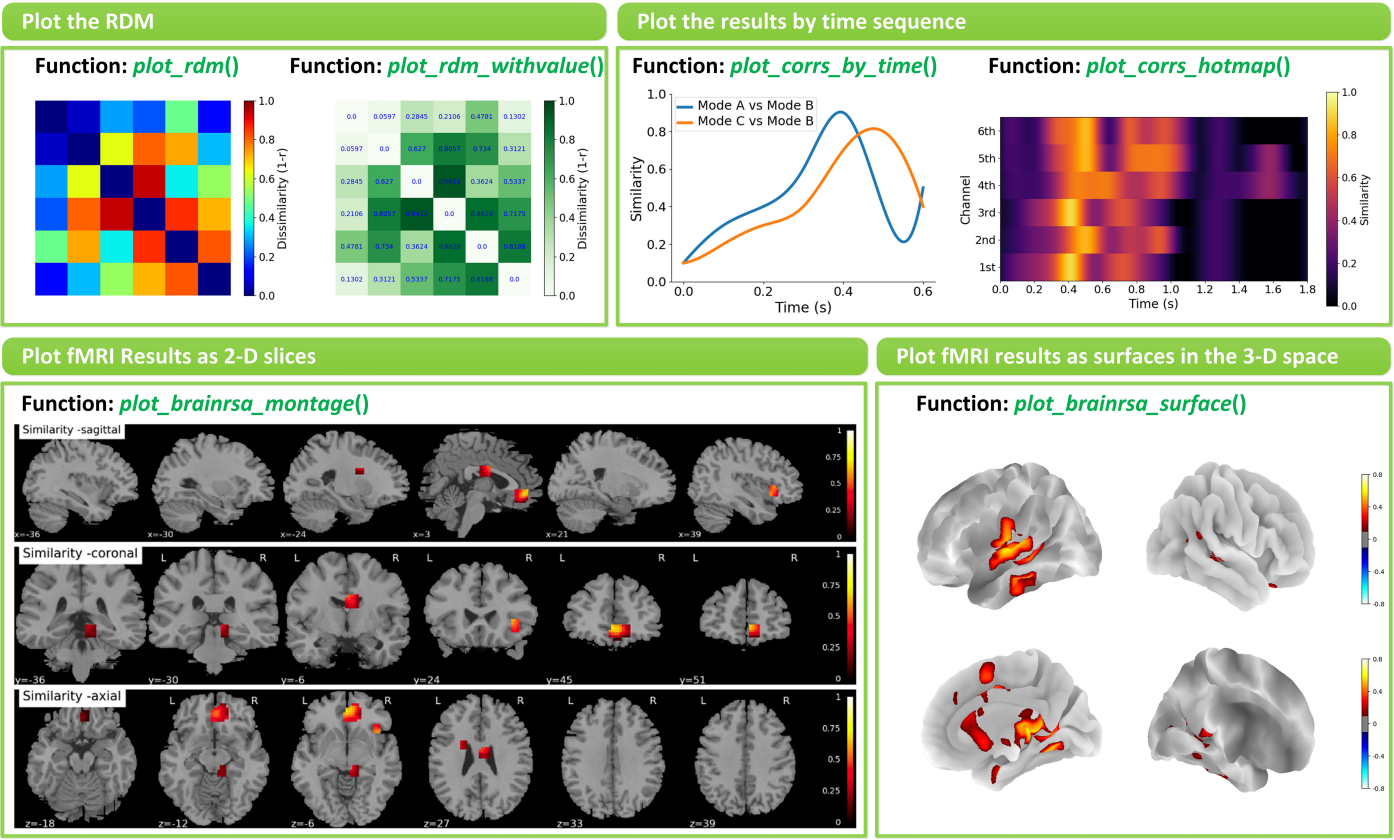
Examples of visualizations implemented in NeuroRA. Left-top: Plot the RDM by function *plot_rdm()* and *plot_rdm_withvalue*(). Right-top: Plot the results by time sequence by function *plot_corrs_by_time*() and *plot_corrs_by_hotmap*(). Left-down: Plot fMRI results as 2-D slices by function *plot_brainrsa_montage*(). Right-down: Plot fMRI results as surface in the 3-D space by function *plot_brainrsa_surface*().

NeuroRA provides several functions to visualize the results in *rsa_plot* module. Some typical features are shown in Figure 7.

The basic option is to visualize RDMs by function *plot_rdm*() or *plot_rdm_withvalue*(). The more advanced option for EEG-like data is to visualize the results across different time points. On the one hand, users can use specific functions, *plot_corrs_by_time*() and *plot_tbytsim_withstats*(), to plot the curve. On the other hand, users can use specific functions, *plot_corrs_hotmap*(), *plot_corrs_hotmap_stats*() (for *r*-values), *plot_stats_hotmap*() (for *t*-values) and *plot_nps_hotmap*() (for NPS), to plot the hotmap. Also, NeuroRA has options for plotting fMRI results on a brain. Users can use functions such as *plot_brainrsa_glass*(), *plot_brainrsa_montage*() and *plot_brainrsa_regions*() to plot fMRI results as 2-D slices, and use *plot_brainrsa_surface*() to plot results as surfaces in the 3-D space. Feature and applicability of functions in *rsa_plot* module are shown in Table 6. The implementation of visualization requires the Pyplot module in the Matplotlib and nilearn package.

**Table 6:** Feature and applicability of functions for plotting results in NeuroRA.

	**Function**	**Feature and Applicability**
for RDM	*plot_rdm*()	Plot the RDM - The input should be an RDM (N_conditions×N_conditions).
	*plot_rdm_withvallue*()	Plot the RDM with values - The input should be an RDM (N_conditions×N_conditions).
for EEG-like	*plot_corrs_by_time*()	Plot the correlation coefficients for different conditions by time sequence - The input should be a matrix (N_conditions×N_time-points) of correlation coefficients.
	*plot_tbytsim_withstats*()	Plot the similarity averaging all subjects by time sequence with statistical results - The input should be a matrix (N_subs×N_time-points) of similarities.
	*plot_corrs_hotmap*()	Plot the hotmap of correlation coefficients for channels/regions by time sequence - The input should be a matrix (N_channels×N_time-points) of correlation coefficients.
	*plot_corrs_hotmap_stats*()	Plot the hotmap of correlation coefficients for channels/regions by time sequence with the significant outline - The input should be a matrix (N_channels×N_time-points) of correlation coefficients and a matrix (N_channels×N_time-points ×2) of *t*-values and *p*-values.
	*plot_stats_hotmap*()	Plot the hotmap of statistical results for channels/regions by time sequence - The input should be a matrix (N_channels×N_time-points ×2) of *t*-values and *p*-values.
	*plot_nps_hotmap*()	Plot the hotmap of NPS for channels/regions by time sequence - The input should be a matrix (N_channels×N_time-points) of similarities.
for fMRI	*plot_brainrsa_glass*()	Plot the 2-D projection of the RSA-results - The input should be the.nii file generated by functions in *neurora.nii_save* module
	*plot_brainrsa_montage*()	Plot the RSA-results by different cuts - The input should be the.nii file generated by functions in *neurora.nii_save* module
	*plot_brainrsa_regions*()	Plot the high-correlation regions of RSA-results by three cuts (frontal, axial, and lateral) - The input should be the.nii file generated by functions in *neurora.nii_save* module
	*plot_brainrsa_surface()*	Plot the RSA-results into a brain surface - The input should be the.nii file generated by functions in *neurora.nii_save* module

**Figure 8:**
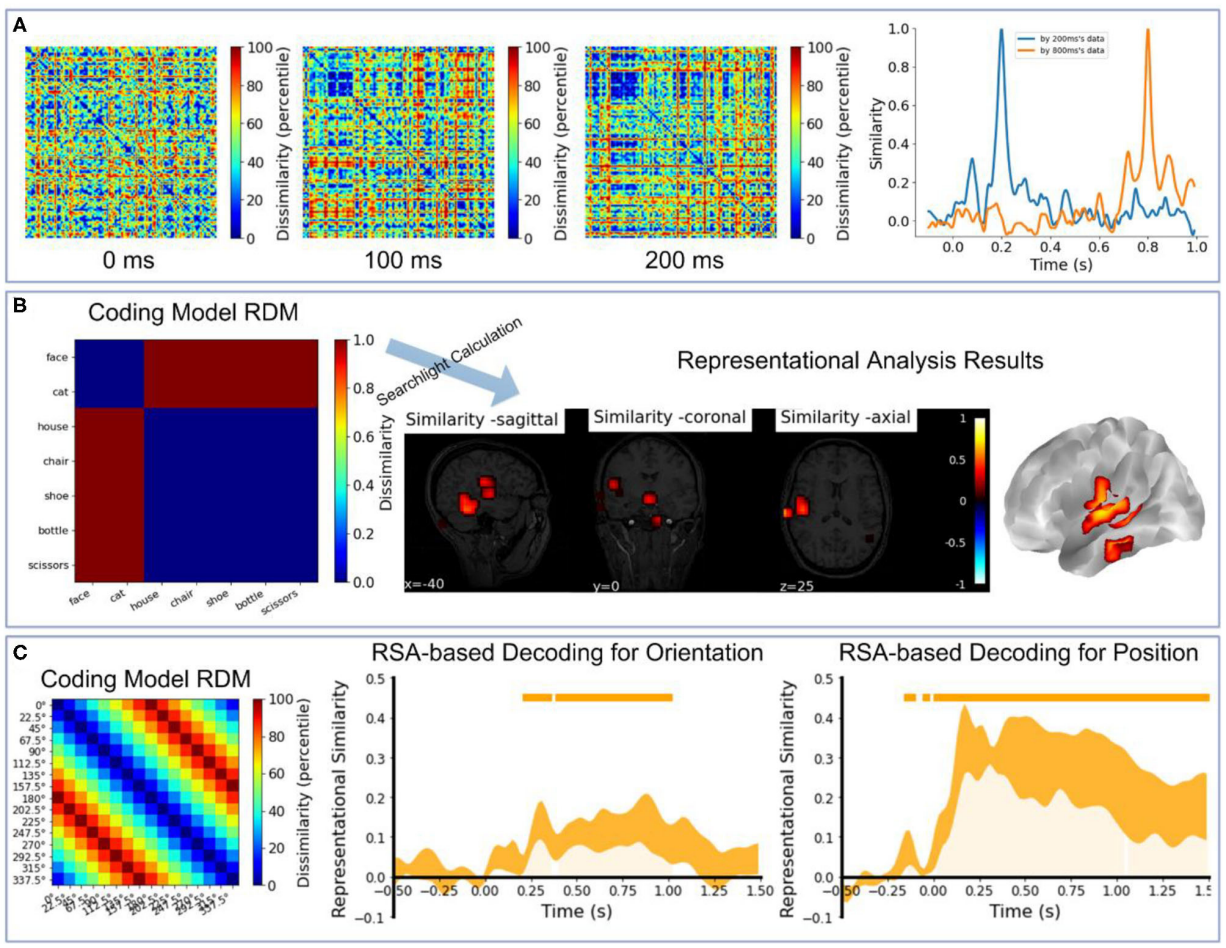
Demo results. **(A)** Left: The RDMs of 0, 100, 200, 300ms based on all 302 channels' MEG data for the first three subjects [data from Cichy et al. (2014)]. Right: Use the neural representations of 200ms and 800ms to calculate the similarities with all time-points' neural representations. **(B)** The searchlight results between an 'animate-inanimate' coding model RDM and searchlight RDMs based on subject2's data [data from Haxby (2001)]. In this coding model RDM, we assume that there are consistent representations among animate objects and inanimate objects. **(C)** The RSA-based decoding results for orientation and position by calculating the correlation coefficients between a coding model RDM and EEG RDMs by time sequence based on the first five subjects' data in experiment 2 [data from Bae and Luck (2019)]. In this coding model RDM, we assume that a large difference between the corresponding two angles corresponds to high dissimilarity, and vice versa. In the two rightmost plots, the small orange rectangles inside the plotting area and orange shadow indicate *p* < 0.05; line width reflects ± SEM.

We also provide several code demos in NeuroRA on the publicly available datasets. The first demo is based on visual-92-categories-task MEG datasets (Cichy et al., 2014). We extracted the first three subjects' data. Figure 8A shows the correlation-based RDMs of three different time-points using NeuroRA [SVM-based RDMs in Cichy et al. (2014) for the first three subjects can be seen in Supplementary Figure 1A] and the temporal similarity results by comparing with the neural representations of 200 and 800 ms. There were more similar neural patterns when participants were viewing human faces (the small blue squares in RDMs), and representations of nearby times were more similar. The second demo is based on the subject2's data in Haxby fMRI datasets (Haxby, 2001). Figure 8 shows the searchlight-based RSA results between an “animate-inanimate” coding model RDM and searchlight RDMs from fMRI data. The results indicated that the temporal cortex was primarily involved in coding information of animate or inanimate. The third demo is based on EEG datasets from a working memory task using NeuroRA (Bae and Luck, 2019). We extracted the first five subjects' event-related potentials (ERP) data. Figure 8C shows the RSA-based decoding results by comparing a coding model RDM and temporal RDMs from EEG data (The temporal SVM-based decoding results of these five subjects can be seen in Supplementary Figure 1). Both orientation and position could be successfully decoding based on ERP data in a visual working memory task. In these demos, user can learn how to use NeuroRA to perform representational analysis and plot the main results, including calculating RDMs from different time points (Figure 8A), correlations over the time series (Figure 8A), searchlight calculation between the brain activities and an “animate-inanimate” coding model (Figure 8B), using the hypothesis-based RDM to fit RDMs based on neural activities by time sequence (Figure 8C) and so on (see more: https://github.com/neurora/NeuroRA/tree/master/demo). These demos contain several critical sections: loading data & preprocessing, calculating RDMs, calculating the neural similarities or similarity matrix, and plotting. Users can download the tutorial on NeuroRA website and find further details.

### User Support

To report any bugs in the code or submit any queries or suggestions about our toolbox, users can use the issue tracker on GitHub: https://github.com/neurora/NeuroRA/issues. We will reply and act accordingly as soon as possible.

## Discussion

RSA provides a novel way of observing big data, which is powerful in the field of cognitive neuroscience. An increasing number of studies have used such multivariate analysis to obtain novel information that could not be observed through univariate analysis (Mahmoudi et al., 2012; Sui et al., 2012; Haxby et al., 2014). More importantly, experimental data obtained from different modalities must be assessed simultaneously, and RSA is a suitable method way to quantitatively compare results from different modalities with distinctive dimensions, resolutions and even obtained from different species (Salmela et al., 2016; Cichy and Pantazis, 2017).

In the present study, we have developed a Python-based toolbox that can perform representation analysis for neural data from many different modalities. Compared with other toolkits or modules that can also implement RSA, our toolbox has a much wider application and more convenient and rich functionalities that users can use tiny codes to conduct not only RSA but also NPS, STPS, ISC, statistical analysis, and visualization, especially for the analysis of multi-modal data and cross-modal comparisons. Moreover, it is open-source, free to use, and cross-platform.

For detailed information on each module and function in our toolbox, including the format of input data, the choice of parameters, and the format of output data, users can refer to our toolbox tutorial. To further understand the specific implementation of each function in the toolbox, people can read the source code directly. If users encounter any problems or difficulties during use, they can consult NeuroRA's tutorials and email our developers.

Although we already implemented the essential functions for representational analysis, there are still several limitations to be addressed in the future. First, NeuroRA is not yet designed to process the raw data. However, users can utilize other toolboxes such as EEGLAB (Delorme and Makeig, 2004), MNE (Gramfort et al., 2013), and Nibabel (Brett et al., 2016) to import data and transfer them into a format fit for NeuroRA. We plan to develop an integrated format conversion function in the next version. Second, there is still significant room for improving the computational performance of NeuroRA, especially in the iterative calculation of fMRI data, which is often slow. This is due to nested loops in the code structure when we need to traverse the data from the entire brain and iterate the random shuffle many times. In the future, we will reduce the time by optimizing functions with GPUs and using some multithreaded methods to accelerate some computing processes. Third, there is no graphical user interface (GUI) right now, which we plan to design and implement based on PyQt in a future version. Users could then obtain the final results by dragging the data file to a specific location in the GUI with the mouse and filling in the relevant parameters. Fourth, object-oriented programming methods can also be applied to our toolkit development. We can build some classes with some public methods requiring the visualization or statistical analysis parameters and some private methods for data management of the multidimensional matrices hidden from the user. Fifth, we need to add some features for the plotting part, such as returning the matplotlib object, assembling subplots and saving them instead of displaying plots on screen only. Sixth, we hope to provide a more straightforward version by streamlining the full analysis workflow building on existing functions. After simplifying the intermediate process, users don't need to call other functions to do extra operations for data transformation. Finally, although we added unit tests in our toolbox, the tests available assess only the shapes of the output corresponding to different inputs rather than check the correctness of the computations. The work to add them will be an important part of NeuroRA's future development.

Through NeuroRA, for the first time, we provide a method for researchers to perform representation analysis with neural data from many different modalities. However, this is only a starting point. With the development of the algorithm and applications for representational analysis, we will include new functionalities, such as using the classification-based decoding accuracy between neural activities under two conditions as the value in an RDM (Cichy et al., 2014; Cichy and Pantazis, 2017; Xie et al., 2020) and automatically generating RDMs for each layer in a deep convolutional neural network, as well as new visualization tools which can plot the space representation of neural activities with t-SNE and show the dynamic representational analysis results. We hope that many exciting findings can be observed through our toolbox, and we would like to collaborate with researchers interested in this tool to improve the toolbox further.

## Information Sharing Statement

NeuroRA is available at https://pypi.org/project/neurora/. The website for NeuroRA is https://neurora.github.io/NeuroRA/, and the tutorial of the toolbox can be download here. Also, users can read online API documentation on https://neurora.github.io/documentation/. The code for our toolbox NeuroRA can be accessed on GitHub: https://github.com/neurora/NeuroRA.

## Data Availability Statement

The original contributions presented in the study are included in the NeuroRA repository (https://github.com/neurora/NeuroRA), further inquiries can be directed to the corresponding author/s.

## Author Contributions

ZL and YK conceived the research, analyze the data, and wrote the paper. ZL coded the toolbox. YK supervised the research.

## Conflict of Interest

The authors declare that the research was conducted in the absence of any commercial or financial relationships that could be construed as a potential conflict of interest.
