# Supplementary material for: NeuroRA: A Python Toolbox of Representational Analysis From Multi-Modal Neural Data
Source: Front Neuroinform. 2020 Dec 23;14:563669. doi: 10.3389/fninf.2020.563669 (PMC7787009; doi:10.3389/fninf.2020.563669)
Supplement: Supplementary file 1 [file Table_1.pdf]

Table S1 Basic structure of inputs and outputs of functions in some key calculation modules in NeuroRA. This table shows the shape of input data, the shape of output data corresponding to different parameter settings and recommended next steps for some key calculation modules, includes *neurora.nps_cal* module, *neurora.isc_cal* module, *neurora.stps_cal* module, *neurora.rdm_cal* module, *neurora.corr_cal* module and *neurora.corr_cal_by_rdm* module. The variable definitions are shown in Table S3.

neurora.nps_cal module

a module for calculating the neural pattern similarity based on neural data

neurora.nps_cal,nps() – to calculate the neural pattern similarity (NPS) for EEG-like data

shape of input data	parameter settings	corresponding shape of output data	recommended next steps
[2^a, *n_subs*, *n_trials*, *n_chls*, *n_ts*]	*sub_opt*=0<br>-return the average results for all subjects	[*n_chls*, *int*((*n_ts*-*time_win*)/*time_step*)+*1*, 2^b]	——
	sub_opt=1<br>-return the results for all subject	[*n_subs*, *n_chls*, *int*((*n_ts*-*time_win*)/*time_step*)+*1*, 2^b]	*neurora.stats_cal.stats*()<br>-to the conduct statistical analysis<br>*neurora.rsa_plot.plot_corrs_hotmap*()<br>-to plot (average the subjects first)

neurora.nps_cal,nps_fmri() – to calculate the neural pattern similarity (NPS) for fMRI data (searchlight)

shape of input data	parameter settings	corresponding shape of output data	recommended next steps
[2^a, *n_subs*, *nx*, *ny*, *nz*]	——	[*n_subs*, *n_x*, *n_y*, *n_z*, 2^b]	*neurora.stats_cal.stats_fmri*()<br>-to the conduct statistical analysis<br>*neurora.nii_save.corr_save_nii*()<br>-to save one subject's results as a .nii file<br>*neurora.nii_save.stats_save_nii*()<br>-to save the statistical results as a .nii file

			(after statistical analysis)

***neurora.nps_cal,nps_fmri_roi*()** – to calculate the neural pattern similarity (NPS) for fMRI data for ROI			
**shape of input data**	**parameter settings**	**corresponding shape of output data**	**recommended next steps**
fmri_data: [2^a, *n_subs*, *nx*, *ny*, *nz*]<br>mask_data: [*nx*, *ny*, *nz*]	——	[*n_subs*, 2^b]	——

neurora.isc_cal **module**

a module for calculating the inter-subject correlation based on neural data

***neurora.isc_cal,isc*()** – to calculate the inter subject correlation (ISC) for EEG-like data			
**shape of input data**	**parameter settings**	**corresponding shape of output data**	**recommended next steps**
[*n_subs*, *n_chls*, *n_ts*]	——	[*n_subs!*/(*2!*×(*n_subs*-2)*!*), *n_chls*, int((*n_ts*-*time_win*)/*time_step*)+*1*, 2^b]	*neurora.stats_cal.stats*()<br>-to the conduct statistical analysis<br>*neurora.rsa_plot.plot_corrs_hotmap*()<br>-to plot (average the subjects first)

***neurora.isc_cal,isc_fmri*()** – to calculate the inter subject correlation (ISC) for fMRI data (searchlight)			
**shape of input data**	**parameter settings**	**corresponding shape of output data**	**recommended next steps**
[*n_ts*, *n_subs*, *nx*, *ny*, *nz*]	——	[*n_ts*, *n_subs!*/(*2!*×(*n_subs*-2)*!*), *n_x*, *n_y*, *n_z*, 2^b]	*neurora.stats_cal.stats_iscfmri*()<br>-to the conduct statistical analysis<br>*neurora.nii_save.stats_save_nii*()<br>-to save the statistical results as a .nii file (after statistical analysis)

***neurora.isc_cal,nps_fmri_roi*()** – to calculate the inter subject correlation (ISC) for fMRI data for ROI			
**shape of input data**	**parameter settings**	**corresponding shape of output data**	**recommended next steps**
fmri_data: [*n_ts*, *n_subs*, *nx*, *ny*, *nz*]<br>mask_data: [*nx*, *ny*, *nz*]	——	[*n_ts*, *n_subs!*/(*2!*×(*n_subs*-2)*!*), 2^b]	——

neurora.stps_cal **module**

a module for calculating the spatiotemporal pattern similarity based on neural data

***neurora.stps_cal,stps*()** – to calculate the spatiotemporal pattern similarity (STPS) for EEG-like data			
**shape of input data**	**parameter settings**	**corresponding shape of output data**	**recommended next steps**
[*n_subs*, *n_trials*, *n_chls*, *n_ts*]	——	[*n_subs*, *8**, *n_chls*, *int*((*n_ts-time_win*)/*time_step*)<br>*+1*]	*neurora.stats_cal.stats_stps*()<br>-to the conduct statistical analysis<br>*neurora.rsa_plot.plot_corrs_hotmap*()<br>-to plot (average the subjects and eight conditions first)

***neurora.stps_cal,stps_fmri*()** – to calculate the spatiotemporal pattern similarity (STPS) for fMRI data (searchlight)			
**shape of input data**	**parameter settings**	**corresponding shape of output data**	**recommended next steps**
[*n_subs*, *n_trials*, *n_x*, *n_y*, *n_z*]	——	[*n_subs*, *8**, *n_x*, *n_y*, *n_z*]	*neurora.stats_cal.stats_stpsfmri*()<br>-to the conduct statistical analysis<br>*neurora.nii_save.stats_save_nii*()<br>-to save the statistical results as a .nii file (after statistical analysis)

***neurora.stps_cal,stps_fmri_roi*()** – to calculate the spatiotemporal pattern similarity (STPS) for fMRI data for ROI			
**shape of input data**	**parameter settings**	**corresponding shape of output data**	**recommended next steps**
fmri_data: [*n_subs*, *n_trials*, *nx*, *ny*, *nz*]<br>mask_data: [*nx*, *ny*, *nz*]	——	[*n_subs*, *8**]	——

neurora.rdm_cal **module**

a module for calculating the RDM based on multimode neural data

***neurora.rdm_cal,bhvRDM*()** – to calculate the RDM(s) for behavioral data			
**shape of input data**	**parameter settings**	**corresponding shape of output data**	**recommended next steps**
[*n_cons*, *n_subs*, *n_trials*]	*sub_opt*=0<br>-return the average RDM for all subjects	[*n_cons*, *n_cons*]	fuctions in *neurora.rdm_corr* module<br>-to calculate the similarity between two RDMs
	sub_opt=1<br>-return the RDMs for all subject	[*n_subs*, *n_cons*, *n_cons*]	functions in *neurora.corr_cal_by_rdm* module<br>-to calculate the similarities between other RDMs and the behavioral RDM<br>*neurora.rsa_plot.plot_rdm*()<br>-to plot one RDM

***neurora.rdm_cal,eegRDM*()** – to calculate the RDM(s) for EEG-like data			
**shape of input data**	**parameter settings**	**corresponding shape of output data**	**recommended next steps**
[*n_cons*, *n_subs*, *n_trials*, *n_chls*,	*sub_opt*=0 & *chl_opt*=0 & *time_opt*=0<br>-average the subjects and trials, and	[*n_cons*, *n_cons*]	fuctions in *neurora.rdm_corr* module<br>-to calculate the similarity between two

n_ts]	return only one RDM		RDMs
	sub_opt=0 & *chl_opt*=0 & *time_opt*=1<br>-average the subjects and trials, calculate for each time-window and return RDMs for each time-window	[*int*((*n_ts*-*time_win*)/*time_step*)+*1*, *n_cons*, *n_cons*]	*neurora.corr_cal_by_rdm.rdms_corr*()<br>-to calculate the similarities between RDMs of EEG-like data and a demo RDM
	sub_opt=0 & *chl_opt*=1 & *time_opt*=0<br>-average the subjects and trials, calculate for each channel and return RDMs for each channel	[*n_chls*, *n_cons*, *n_cons*]	*neurora.rsa_plot.plot_rdm*()<br>-to plot one RDM
	sub_opt=0 & *chl_opt*=1 & *time_opt*=1<br>-average the subjects and trials, calculate for each channel and each time-window, and return RDMs for each channel and each time-window	[*n_chls*, *int*((*n_ts*-*time_win*)/*time_step*)+*1*, *n_cons*, *n_cons*]	
	sub_opt=1 & *chl_opt*=0 & *time_opt*=0<br>-average the trials, calculate for each subject, return RDMs for each subject	[*n_subs*, *n_cons*, *n_cons*]	
	sub_opt=1 & *chl_opt*=0 & *time_opt*=1<br>-average the trials, calculate for each subject and each time-window, and return RDMs for each subject and each time-window	[*n_subs*, *int*((*n_ts*-*time_win*)/*time_step*)+*1*, *n_cons*, *n_cons*]	
	sub_opt=1 & *chl_opt*=1 & *time_opt*=0<br>-average the trials, calculate for each subject and each channel and return RDMs for each subject and each channel	[*n_subs*, *n_chls*, *n_cons*, *n_cons*]	
	sub_opt=1 & *chl_opt*=1 & *time_opt*=1	[*n_subs*, *n_chls*, *int*((*n_ts*-	

	-average the trials, calculate for each subject, each channel and each time-window, and return RDMs for each subject, each channel and each time-window	*time_win*)/*time_step*)+*1*, *n_cons*, *n_cons*]	

***neurora.rdm_cal,fmriRDM*()** – to calculate the RDM(s) for fMRI data (searchlight)

**shape of input data**	**parameter settings**	**corresponding shape of output data**	**recommended next steps**
[*n_cons*, *n_subs*, *nx*, *ny*, *nz*]	*sub_opt*=0<br>-calculate for each subject, and return the average RDMs for all subjects	[*n_x*, *n_y*, *n_z*, *n_cons*, *n_cons*]	fuctions in *neurora.rdm_corr* module<br>-to calculate the similarity between two RDMs<br>*neurora.corr_cal_by_rdm.fmrirdms_corr*()<br>-to calculate the similarities between RDMs of fMRI data and a demo RDM<br>*neurora.rsa_plot.plot_rdm*()<br>-to plot one RDM
	sub_opt=1<br>-calculate for each subject, and return the RDMs for all subject	[*n_subs*, *n_x*, *n_y*, *n_z*, *n_cons*, *n_cons*]	

***neurora.rdm_cal,fmriRDM_roi*()** – to calculate the RDM(s) for fMRI data (for ROI)

**shape of input data**	**parameter settings**	**corresponding shape of output data**	**recommended next steps**
fmri_data: [*n_subs*, *n_trials*, *nx*, *ny*, *nz*]<br>mask_data: [*nx*, *ny*, *nz*]	*sub_opt*=0<br>-calculate for each subject, and return the average RDM for all subjects	[*n_cons*, *n_cons*]	——
	sub_opt=1<br>-calculate for each subject, and return the RDMs for all subject	[*n_subs*, *n_cons*, *n_cons*]	

neurora.corr_cal **module**

a module for calculating the similarity between two different modes' data

***neurora.corr_cal,bhvANDeeg_corr*()** – to calculate the similarity between behavioral data and EEG-like data

shape of input data	parameter settings	corresponding shape of output data	recommended next steps
bhv_data: [*n_cons*, *n_subs*, *n_trials*]<br>eeg_data: [*n_cons*, *n_subs*, *n_trials*, *n_chls*, *n_ts*]	*sub_opt*=0 & *chl_opt*=0 & *time_opt*=0<br>-calculate one RDM for behavioral data and one RDM for EEG-like data, return the similarity between these two RDMs	[*2*[b]]	When *sub_opt*=1:<br>*neurora.stats_cal.stats*()<br>-to the conduct statistical analysis
	sub_opt=0 & *chl_opt*=0 & *time_opt*=1<br>-calculate one RDM for behavioral data and multiple RDMs for each time-window for EEG-like data, and return the similarity between behavioral RDM and EEG-like data's RDMs	[*int*((*n_ts*-*time_win*)/*time_step*)+*1*, *2*[b]]	
	sub_opt=0 & *chl_opt*=1 & *time_opt*=0<br>-calculate one RDM for behavioral data and multiple RDMs for each channel for EEG-like data, and return the similarity between behavioral RDM and EEG-like data's RDMs	[*n_chls*, *2*[b]]	
	sub_opt=0 & *chl_opt*=1 & *time_opt*=1<br>-calculate one RDM for behavioral data and multiple RDMs for each channel and each time-window for EEG-like data, and return the similarity between behavioral RDM and EEG-like data's RDMs	[*n_chls*, *int*((*n_ts*-*time_win*)/*time_step*)+*1*, *2*[b]]	

	sub_opt=1 & *chl_opt*=0 & *time_opt*=0<br>-calculate multiple RDMs for each subject for behavioral data and multiple RDMs for each subject for EEG-like data, and return the similarity between behavioral RDMs and EEG-like data's RDMs	[*n_subs*, *2*[b]]
	sub_opt=1 & *chl_opt*=0 & *time_opt*=1<br>-calculate multiple RDMs for each subject for behavioral data and multiple RDMs for each subject and each time-window for EEG-like data, and return the similarity between behavioral RDMs and EEG-like data's RDMs	[*n_subs*, *int*((*n_ts-time_win*)/*time_step*)+*1*, *2*[b]]
	sub_opt=1 & *chl_opt*=1 & *time_opt*=0<br>-calculate multiple RDMs for each subject for behavioral data and multiple RDMs for each subject and each channel for EEG-like data, and return the similarity between behavioral RDMs and EEG-like data's RDMs	[*n_subs*, *n_chls*, *2*[b]]
	sub_opt=1 & *chl_opt*=1 & *time_opt*=1<br>-calculate multiple RDMs for each subject for behavioral data and multiple RDMs for each subject, each channel and each time-window for EEG-like data, and return the similarity between behavioral RDMs and EEG-like data's RDMs	[*n_subs*, *n_chls*, *int*((*n_ts-time_win*)/*time_step*)+*1*, *2*[b]]

***neurora.corr_cal,bhvANDfmri_corr*()** – to calculate the similarity between behavioral data and fMRI data (searchlight)

shape of input data	parameter settings	corresponding shape of output data	recommended next steps
bhv_data: [*n_cons*, *n_subs*, *n_trials*]<br>fmri_data: [*n_cons*, *n_subs*, *nx*, *ny*, *nz*]	*sub_result*=0<br>-calculate for each subject, and return the average similarities for all subjects	[*n_x*, *n_y*, *n_z*, 2^b]	When *sub_result*=1:<br>*neurora.stats_cal.stats_fmri*()<br>-to the conduct statistical analysis
	sub_result=1<br>-calculate for each subject, and return the similarities for all subject	[*n_subs*, *n_x*, *n_y*, *n_z*, 2^b]	

***neurora.rdm_cal,eegANDfmri_corr*()** – to calculate the similarity between EEG-like data for fMRI data (for ROI)

shape of input data	parameter settings	corresponding shape of output data	recommended next steps
eeg_data: [*n_cons*, *n_subs*, *n_trials*, *n_chls*, *n_ts*]<br>fmri_data: [*n_cons*, *n_subs*, *nx*, *ny*, *nz*]	*chl_opt*=1 & *sub_result*=1<br>-calculate multiple RDMs for EEG-like data for each channel and each subject and RDMs for fMRI data for each subject, return the similarities between EEG-like RDMs and fMRI RDMs for each subject	[*n_subs*, *n_chls*, *n_x*, *n_y*, *n_z*, 2^b]	When *sub_result*=1:<br>*neurora.stats_cal.stats*()<br>-to the conduct statistical analysis
	chl_opt=1 & *sub_result*=0<br>-calculate multiple RDMs for EEG-like data for each channel and each subject and RDMs for fMRI data for each subject, return the averaged similarities between EEG-like RDMs and fMRI RDMs for all subjects	[*n_chls*, *n_x*, *n_y*, *n_z*, 2^b]	
	chl_opt=0 & *sub_result*=1<br>-calculate multiple RDMs for EEG-like	[*n_subs*, *n_x*, *n_y*, *n_z*, 2^b]	

	data for each subject and RDMs for fMRI data for each subject, return the averaged similarities between EEG-like RDMs and fMRI RDMs for all subjects		
	chl_opt=0 & *sub_result*=0<br>-calculate one RDM for EEG-like data and multiple RDMs after averaging subjects for EEG-like data, and return the similarities between EEG-like RDM and fMRI data's RDMs	[*n_x*, *n_y*, *n_z*, 2^b]	

***neurora.corr_cal_by_rdm* module**

a module for calculating the similarity between two different modes' data

***neurora.corr_cal_by_rdm,rdms_corr*()** – to calculate the similarity between RDMs of EEG-like data and a demo RDM

shape of input data	parameter settings	corresponding shape of output data	recommended next steps
demo_rdm: [*n_cons*, *n_cons*]<br>eeg_rdms: [*n_cons*, *n_cons*] or [*n1*, *n_cons*, *n_cons*] or [*n1*, *n2*, *n_cons*, *n_cons*] or [*n1*, *n2*, *n3*, *n_cons*, *n_cons*]	——	[2^b] or [*n1*, 2^b] or [*n1*, *n2*, 2^b] or [*n1*, *n2*, *n3*, 2^b]	When *sub_opt*=1:<br>*neurora.stats_cal.stats*()<br>-to the conduct statistical analysis

***neurora.corr_cal_by_rdm,fmrirdms_corr*()** – to calculate the similarity between fMRI searchlight RDMs and a demo RDM

shape of input data	parameter settings	corresponding shape of output data	recommended next steps

demo_rdm: [*n_cons*, *n_cons*]<br>fmri_rdms: [*nx*, *ny*, *nz*, *n_cons*, *n_cons*]	——	[*nx*, *ny*, *nz*, 2^b]	When *sub_result*=1:<br>*neurora.stats_cal.stats*()<br>-to the conduct statistical analysis
