# Supplementary material for: NeuroRA: A Python Toolbox of Representational Analysis From Multi-Modal Neural Data
Source: Front Neuroinform. 2020 Dec 23;14:563669. doi: 10.3389/fninf.2020.563669 (PMC7787009; doi:10.3389/fninf.2020.563669)
Supplement: Supplementary file 2 [file Table_2.pdf]

Table S2 Basic structure of inputs and outputs of functions in *stats_cal* module in NeuroRA. The variable definitions are shown in Table S3.

**stats()**<br>– to conduct the statistical analysis for results of EEG-like data	
shape of input data:<br>[*n_subs*, *n_chls*, *n_ts*, 2^b]	shape of output data:<br>[*n_cons*, *n_cons*]
**stats_fmri()**<br>– to conduct the statistical analysis for results of fMRI data (searchlight)	
shape of input data:<br>[*n_subs*, *n_chls*, *n_ts*, 2^b]	shape of output data:<br>[*n_cons*, *n_cons*]
**stats_iscfmri()**<br>– to conduct the statistical analysis for results of fMRI data (ISC searchlight)	
shape of input data:<br>[*n_ts*, *n_subs!/(2!×(n_subs-2)!)*, *n_x*, *n_y*, *n_z*, 2^b]	shape of output data:<br>[*n_ts*, *n_x*, *n_y*, *n_z*, 2^b]
**stats_stps()**<br>– to conduct the statistical analysis for results of EEG-like data (for STPS)	
shape of input data:<br>*corrs1*: [*n_subs*, *n_chls*, *n_ts*]<br>*corrs2*: [*n_subs*, *n_chls*, *n_ts*]	shape of output data:<br>[*n_chls*, *n_ts*, 2^b]
**stats_stpsfmri()**<br>– to conduct the statistical analysis for results of fMRI data (searchlight)	
shape of input data:<br>*corrs1*: [*n_subs*, *n_x*, *n_y*, *n_z*]<br>*corrs2*: [*n_subs*, *n_x*, *n_y*, *n_z*]	shape of output data:<br>[*n_x*, *n_y*, *n_z*, 2^b]
