# Supplementary material for: NeuroRA: A Python Toolbox of Representational Analysis From Multi-Modal Neural Data
Source: Front Neuroinform. 2020 Dec 23;14:563669. doi: 10.3389/fninf.2020.563669 (PMC7787009; doi:10.3389/fninf.2020.563669)
Supplement: Supplementary file 3 [file Table_3.pdf]

Table S3 Definitions of the variables in Table S1 and Table S2.

Variable	Definition
2^a	2 conditions (to calculate the NPS)
n_subs	the number of subjects
n_trials	the number of trials
n_chls	the number of channels
n_ts	the number of time-points
time_window	the number of time-points in each time interval for the calculation
time_step	the number of time-points in each time step for calculation
2^b	2 values, including an *r*-value and a *p*-value
nx, *ny*, *nz*	the size of fMRI-img
n_x, *n_y*, *n_z*	the number of calculation unit for searchlight along the x, y, z axis
*8**	8 conditions for comparing (after calculating the STPS)
n_cons	the number of conditions
