# Supplementary material for: NeuroRA: A Python Toolbox of Representational Analysis From Multi-Modal Neural Data
Source: Front Neuroinform. 2020 Dec 23;14:563669. doi: 10.3389/fninf.2020.563669 (PMC7787009; doi:10.3389/fninf.2020.563669)
Supplement: Supplementary file 4 [file Presentation_1.pdf]

Supplementary instructions for data conversion

For EEG/MEG data

Users can use MATLAB toolbox such as EEGLab (http://sccn.ucsd.edu/eeglab/) to do preprocessing and obtain *.mat* files, then use SciPy (https://www.scipy.org) to load EEG data (*.mat*) as ndarray-type data. Sample codes:

```
>>> import scipy.io as sio
>>> data = sio.loadmat(filename)[“data”])
```

Or users can use MNE (https://mne-tools.github.io) to do preprocessing and return ndarray-type data. Sample codes:

```
>>> # here epoch should be an Epoch object in MNE-Python
>>> data = epoch.get_data()
```

Also, for EEG data, users can use Neo (Garcia et al., 2014) (https://neuralensemble.org/neo/) to preprocess and return ndarray-type data. See more detail in Neo io module, and it provides many methods for reading different formats from different EEG acquisition systems.

For fMRI data

We strongly recommend users to use Nibabel (https://nipy.org/nibabel/) to load fMRI data as ndarray-type data. Sample codes:

```
>>> import nibabel as nib
>>> data = nib.load(fmrifilename).get_fdata()
```

For fNIRS data

For raw data from device, users can use Numpy (http://www.numpy.org) to load fNIRS data (*.txt* or *.csv*) as ndarray-type data. Sample codes:

```
>>> import numpy as np
>>> # load fNIRS data of .txt file as ndarray
>>> data = np.loadtxt(txtfilename)
>>> # load fNIRS data of .csv file as ndarray
>>> data = np.loadtxt(csvfilename, delimiter, usecols, unpack)
```

For other sources of neuroelectrophysiological data

Users can use Brainstorm (https://neuroimage.usc.edu/brainstorm/) to

preprocess and obtain .mat files and then use SciPy to load ECoG data (*.mat*) as ndarray-type data.

Or users can use Neo (https://neuralensemble.org/neo/) to do preprocessing and return ndarray-type data. See more detail in Neo io module, and it provides many methods for reading different formats from different neuroelectrophysiology acquisition systems.

Also, users can use pyABF (https://github.com/swharden/pyABF) for Axon system, to load electrophysiology data (*.abf*) as ndarray-type data. Sample codes:

```
>>> import pyabf
>>> # the electrophysiology data file name with full address
>>> abf = pyabf.ABF("demo.abf")
>>> # access sweep data
>>> abf.setSweep(sweepNumber, channel)
>>> # get sweep data with sweepY
>>> data = abf.sweepY
```

Notes

Two functions, *NumPy.reshape*() & *NumPy.transpose*(), are recommended for further data transformation.
