# Supplementary material for: NeuroRA: A Python Toolbox of Representational Analysis From Multi-Modal Neural Data
Source: Front Neuroinform. 2020 Dec 23;14:563669. doi: 10.3389/fninf.2020.563669 (PMC7787009; doi:10.3389/fninf.2020.563669)
Supplement: Supplementary file 5 [file Presentation_2.pdf]

## Supplementary information for implementation of analysis methods

### Neural Pattern Similarity (NPS)

NPS is used to compare the neural activities under two different conditions. Users can input the data under two different conditions to functions in *neurora.nps_cal* module to obtain the similarity result. Two conditions can be two different task conditions in an experiment, such as viewing two kinds of stimuli (e.g., response to faces vs. response to houses in Haxby et al., 2001). In NeuroRA, we convert multidimensional neural activities into vectors and then calculate the similarities (Pearson Correlation) between two vectors corresponding to two different conditions. To be more specific, we provide multiple functions to calculate NPS for EEG-like data and fMRI data. For EEG-like data (using *nps*() function), users can set the time-window (the parameter: *time_win*) and time-step (the parameter: *time_step*) for customized computing. We calculate NPS for each subject. If *sub_opt*=0, our functions will return the average RDM(s) for all subjects. If *sub_opt*=1, our functions will return RDM(s) for each subject. For instance, if user sets *time_opt*=1, *time_win*=10, and *time_step*=5, the function will calculate the NPS results based on each time-window consisting of ten time-points, and the time step is a time interval consisting of 5 time-points between two time-windows for calculation. For fMRI data, users can set the calculation unit for searchlight calculation (using *nps_fmri*() function) or calculate the result from an ROI by a given mask matrix (using *nps_fmri_roi*() function). NPS is calculated for each subject, and users can receive all subjects' NPS results.

### Spatiotemporal Pattern Similarity (STPS)

STPS is an improvement in NPS for a memory and learning task, and it reflects the representational similarity across different space and time points. In the experiment that researchers further want to calculate STPS, participants are asked to view stimuli under different conditions, and each stimulus is repeated several times (Xue et al., 2010; Lu et al., 2015). STPS can be used to track the representation of this learning process. In NeuroRA, *stps_cal* module is provided to calculate STPS. Users need to input data of neural activities with label information for each trial. Two kinds of labels, labels for the category of the item in each trial and labels for the subject remembering or forgetting the item in each trial, need to input. For STPS computing, we extract the data vector for each trial and calculate the correlation matrix among all the trials. Then we extract the correlation coefficients (Pearson Correlation) from the correlation matrix under different conditions based on the labels users input. Also, we provide multiple functions to calculate STPS for both EEG-like data and fMRI data. For EEG-like data (using *stps*() function), users can set the time-window

(the parameter: *time_win*) and time-step (the parameter: *time_step*) for customized computing. For instance, if user sets *time_win*=10 and *time_step*=5, the function will calculate the STPS results based on each time-window consisting of ten time-points, and the time step is a time interval consisting of 5 time-points between two time-windows for calculation. For fMRI data, users can set the calculation unit for searchlight calculation (using *stps_fmri*() function) or calculate the result from an ROI by a given mask matrix (using *stps_fmri_roi*() function). For both EEG-like and fMRI data, STPS is calculated for each subject, and users can receive all subjects' STPS results.

**Inter-Subject Correlation (ISC)**

ISC is used to calculate the similarity of brain activities among multiple subjects (Hasson et al., 2004). In NeuroRA, *isc_cal* module is provided to calculate ISC. We convert multidimensional neural activities into vectors and calculate the correlation coefficients (Pearson Correlation) of all pairs of subjects. Consistent with NPS and STPS, users can calculate ISC for both EEG-like data and fMRI data using NeuroRA. For EEG-like data (using *isc*() function), users can set the time-window (the parameter: *time_win*) and time-step (the parameter: *time_step*) for customized computing. For instance, if user sets *time_win*=10 and *time_step*=5, the function will calculate the ISC results based on each time-window consisting of ten time-points, and the time step is a time interval consisting of 5 time-points between two time-windows for calculation. For fMRI data, users can set the calculation unit for searchlight calculation (using *isc_fmri*() function) or calculate the result from an ROI by a given mask matrix (using *isc_fmri_roi*() function). For both EEG-like and fMRI data, ISC is calculated for each subject, and users can receive all subjects' ISC results.

**Representational Similarity Analysis (RSA)**

*Calculating RDMs*: NeuroRA provided *rdm_cal* module to calculate RDMs for data from different modalities (using *bhvRDM*() function for behavioral data, *eegRDM*() for EEG-like data, *fmriRDM*() and *fmriRDM_roi*() functions for fMRI data). In all functions in *rdm_cal* module, we calculate RDM(s) for each subject. If *sub_opt*=0, our functions will return the average RDM(s) for all subjects. If *sub_opt*=1, our functions will return RDM(s) for each subject.

Also, there are several alternative methods such as correlation distance, Euclidean distance, and Mahalanobis distance to construct an RDM. Users can set the parameter *method* to choose which method they like. We reshaped activations under different conditions into feature vectors. For correlation distance (*method*= "*correlation*"), values in each RDM are calculated the 1 – Pearson rho between vectors under two corresponding conditions. For Euclidean distance (*method*= "*euclidean*"), values in each RDM are calculated

the Euclidean distance between two vectors, which means the distance between two points in high-dimensional space. Then we rescale all values to the range [0, 1]. For Mahalanobis distance (*method*= "*mahalanobis*"), values in each RDM are calculated the Mahalanobis distance between vectors under two corresponding conditions and are rescaled to the range [0, 1].

Specifically, for EEG-like data (using *eegRDM*() function), we average the trials under each condition first. For channel-based computing (*chl_opt*=1), we calculate RDMs for each channel and return each channel's results. For time-based computing (*time_opt*=1), we calculate RDMs for each time-window based on parameters (*time_win* and *time_step*) user sets. For instance, if user sets *time_opt*=1, *time_win*=10, and *time_step*=5, the function will calculate the RDMs based on each time-window consisting of ten time-points, and the time step is a time interval consisting of 5 time-points between two time-windows for calculation.

*Calculating representational similarity*: NeuroRA provides three modules to calculate representational similarity. In *rdm_corr* module, functions are applied to calculate the similarity between two RDMs. Users can choose different functions to get the appropriate similarity index. We first extract the values of the upper half of two RDMs and reformed them into two vectors. *rdm_correlation_spearman*(), *rdm_correlation_pearson*() and *rdm_correlation_kendall*() are used to calculate Spearman correlation, Pearson correlation and Kendall's tau correlation, respectively. These three functions return an *r*-value and a *p*-value. The other two functions, *rdm_similarity*() and *rdm_distance*(), are applied to calculate the cosine similarity and Euclidean distance between two RDMs, respectively. These two functions return the distance.

In *corr_cal_by_rdm* module, we provide functions to conduct complex calculations based on multiple RDMs. Users input RDMs from two different modalities, behavioral RDM and EEG-like RDMs (using *rdms_corr*() function) or behavioral RDM and fMRI RDMs (using *fmrirdms_corr*() function). In these processes, we essentially do the calculation by calling functions in *rdm_corr* module. Setting different values for the parameter method, such as "spearman", "pearson", "kendall", "similarity" and "distance", corresponds to different calculation methods for computing the similarity among RDMs.

In *corr_cal* module, we provide functions to obtain similarities from data from different modalities (using *bhvANDeeg_corr*() function for behavioral and EEG-like data, using *bhvANDfmri_corr*() function for behavioral and fMRI data, using *eegANDfmri_corr*() function for EEG-like and fMRI data). The implementation of this module is combining *rdm_cal* module and *corr_cal_by_rdm* module. We use *rdm_cal* module to calculate RDMs based

on neural data. Then we use *corr_cal_by_rdm* module to get the representational similarity between the two modalities.

**Statistical Analysis**

Statistical analysis is a necessary step after conducting various representational analyses. Due to different shapes of outputs corresponding to different computing, NeuroRA provides necessary functions to realize statistical analysis for different representational analysis results in *stats_cal* module (see Table S2). In all functions, all samples in input maps of correlation coefficients from subjects are tested against zero for significance based on *stats* module in Scipy. Accordingly, these functions will return a *t*-value map and a *p*-value map reflecting the statistical results.
