# Supplementary material for: NeuroRA: A Python Toolbox of Representational Analysis From Multi-Modal Neural Data
Source: Front Neuroinform. 2020 Dec 23;14:563669. doi: 10.3389/fninf.2020.563669 (PMC7787009; doi:10.3389/fninf.2020.563669)
Supplement: Supplementary file 6 [file Presentation_3.pdf]

Supplementary References

Bae, G. Y., and Luck, S. J. (2019). Dissociable decoding of spatial attention and working memory from EEG oscillations and sustained potentials. *J. Neurosci*. 38, 409–422. doi: 10.1523/JNEUROSCI.2860-17.2017

Cichy, R. M., Pantazis, D., and Oliva, A. (2014). Resolving human object recognition in space and time. *Nat. Neurosci*. 17, 455–462. doi: 10.1038/nn.3635

Garcia, S., Guarino, D., Jaillet, F., Jennings, T., Pröpper, R., Rautenberg, P. L., et al. (2014). Neo: an object model for handling electrophysiology data in multiple formats. *Front. Neuroinform*. 8:10. doi: 10.3389/fninf.2014.00010

Hasson, U., Nir, Y., Levy, I., Fuhrmann, G., and Malach, R. (2004). Intersubject synchronization of cortical activity during natural vision. *Science*. 303, 1634–1640. doi: 10.1126/science.1089506

Haxby, J. V. (2001). Distributed and overlapping representations of faces and objects in ventral temporal cortex. *Science*. 293, 2425–2430. doi: 10.1126/science.1063736

Lu, Y., Wang, C., Chen, C., and Xue, G. (2015). Spatiotemporal neural pattern similarity supports episodic memory. *Curr. Biol*. 25, 780–785. doi: 10.1016/j.cub.2015.01.055

Xue, G., Dong, Q., Chen, C., Lu, Z., Mumford, J. A., and Poldrack, R. A. (2010). Greater neural pattern similarity across repetitions is associated with better memory. *Science*. 330, 97–101. doi: 10.1126/science.1193125
