# Supplementary material for: NeuroRA: A Python Toolbox of Representational Analysis From Multi-Modal Neural Data
Source: Front Neuroinform. 2020 Dec 23;14:563669. doi: 10.3389/fninf.2020.563669 (PMC7787009; doi:10.3389/fninf.2020.563669)
Supplement: Supplementary file 7 [file Image_1.pdf]

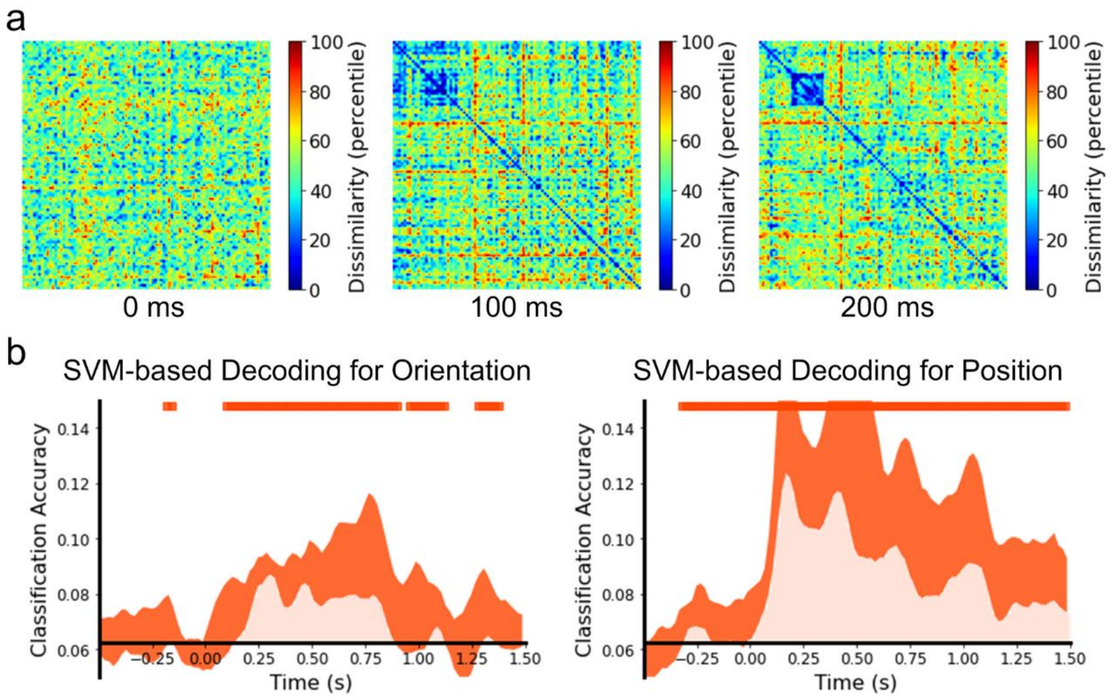

Figure S1 SVM-based results compared with results using NeuroRA in Figure 8a and Figure 8c. (a) RDMs of the first three subjects, calculated by SVM-based classification (Cichy et al., 2014) and plotted by *neurora.rsa_plot.plot_rdm*(). Cichy et al. used the classification accuracy of two categories as the value corresponding to two different conditions in RDM. (b) Orientation and position decoding results of the first five subjects in experiment 2 by SVM-based classification (Bae and Luck, 2019). The results were reproduced by codes rewritten in Python. In the two rightmost plots, the small red rectangles inside the plotting area and red shadow indicate $p < 0.05$; line width reflects ± SEM.
